# Particle Size Measurement of Organic Nanocarriers: A Case Study Using Polyacrylate‐Based Polymer Nanocarriers

**DOI:** 10.1002/smsc.70415

**Published:** 2026-10-05

**Authors:** Dona Manayath, Monica Granetto, Tiziana Tosco, Jadranka Travas‐Sejdic, Erin M. Leitao, Melanie Kah

**Affiliations:** ^1^ School of Chemical Sciences University of Auckland Auckland New Zealand; ^2^ The MacDiarmid Institute for Advanced Materials and Nanotechnology Wellington New Zealand; ^3^ Department of Environmental, Land and Infrastructure Engineering (DIATI) Politecnico di Torino Turin Italy; ^4^ School of Environment University of Auckland Auckland New Zealand

**Keywords:** azoxystrobin, characterization, DLS, hydrodynamic diameter, nanoparticles, OECD guidelines, polydispersity

## Abstract

This study evaluates the sensitivity, detection limits, and applicability of size measurement techniques to polyacrylate‐based polymer nanocarriers (PNCs) in a hydrated state. Dynamic light scattering (DLS), particle tracking analysis (PTA), cryo‐transmission electron microscopy (cryo‐TEM), scanning electron microscopy (SEM), and differential centrifugal sedimentation (DCS) are applied to commercial and laboratory‐scale PNCs to assess the effect of UV‐mediated crosslinking, as well as the impact of active substances (ASs) and commercial dispersants on size distribution. Polydispersity and low refractive index make DLS unreliable. PTA does not detect PNCs <50 nm and requires adapting concentrations to each PNC, challenging comparisons. DCS captures polydispersity but could not detect PNCs <50 nm either. Importantly, DLS, PTA, and DCS assume spherical geometry, but commercial PNCs are unlikely to be spherical, thus seriously challenging data interpretation. Cryo‐TEM detects PNCs <50 nm and delivers the most reliable size distribution. Large differences among the PNCs tested warn against the use of model PNCs and read across when conducting risk assessment. Our results confirm the challenges and inadequacies in existing approaches and call for more research to develop multimethod approaches and adaptation of OECD guidelines to characterize PNCs, which are increasingly used across various sectors.

## Graphical abstract

Sensitivity, detection limits and applicability of size measurement techniques to polymer nanocarriers (PNCs) are investigated. PTA and DCS do not detect PNCs <50 nm and PTA requires adapting concentrations to each PNC, challenging comparisons. Spherical assumptions challenge interpretation by DLS, PTA, and DCS. Cryo‐TEM detects PNCs <50 nm. Large differences among PNCs challenge use of model particles and read‐across approaches.

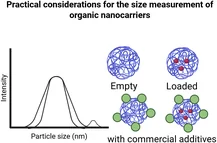

## 1 Introduction

According to the European Food Safety Authority (EFSA), a nanomaterial is defined as a material that contains particles in an unbound state or as an aggregate or agglomerate with 50% or more particles in the size distribution range of 1–100 nm [1]. For human health regulatory purposes, nanomaterials can also include particles with size above 100 nm, while retaining their nanoscale properties [2]. Therefore, polymer nanocarriers (PNCs) include a diverse range of soft nanoparticles with particle sizes ranging from 1 to 1000 nm [3]. PNCs are used as delivery systems for the loaded active substances (ASs) such as vitamins, pharmaceuticals, or agrochemicals [3]. The accurate and reproducible characterization of the chemical and physical properties of empty and loaded PNCs is essential to assess the potential risks associated with PNC‐containing products used in the food, pharmaceutical, and agricultural sectors [4, 5, 6, 7, 8]. Among the different characterization parameters, particle size distribution and polydispersity (PDI) are particularly important as these two parameters determine the uniformity, efficacy, invitro/invivo behavior, and physicochemical stability of PNC suspensions over extended storage periods [9, 10]. PDI is a dimensionless quantity that measures the nonuniformity in the size distribution of the empty and loaded PNCs, with values less than 0.05 from dynamic light scattering (DLS) experiments indicating uniform particle size distribution or monodispersity [9]. The size distribution and PDI directly influence particle interactions with biological systems, such as cellular uptake, biodistribution, and excretion, as well as environmental exposure [11]. In nanomedicine, particles <5 nm undergo renal clearance, and particles <200 nm are removed by the spleen, whereas particles >200 nm are retained in the liver endothelium and particles >2–5 μm are accumulated in the lung capillaries [12]. PNCs that are larger than the cellular membrane thickness (>50 nm) need more time to cross the cellular membrane through membrane wrapping [13, 14, 15]. Therefore, particles <200 nm are typically considered advantageous for therapeutic delivery as they support membrane crossing and biological clearance [16]. For screening purposes, a larger size cutoff may be considered. For instance, the EFSA adopted a size cutoff of 500 nm for nanoparticles, based on the evidence of gastrointestinal uptake at 250 nm, along with an uncertainty factor of 2 to account for the analytical limitations [17]. The limitations include the particle size range that can be detected, quantified, and converted into a number‐based distribution. EFSA adopted a threshold of 500 nm to achieve a high level of consumer protection. For instance, a ≤10% number‐based distribution of particles below 500 nm is considered indicative of negligible nanoscale exposure within the range of 1–100 nm, for which a conventional risk assessment is generally sufficient. Particle size directly affects the barrier crossing properties and biological fate of PNCs, and accurately determining the size distribution of PNCs is essential for higher‐tier risk assessment [18].

In 2006, the Organization for Economic Co‐operation and Development (OECD) working party on manufactured nanomaterials was established to advance guidance for nanosafety in regulatory research. Over the last decade, significant progress has been made in adapting existing or developing new test guidelines for inorganic nanomaterials [19, 20, 21, 22, 23]. The OECD Test Guideline 125, adopted in 2022, focuses on the particle size distribution of different inorganic nanomaterials, including metals, metal oxides, and carbon‐containing inorganic nanomaterials such as single and multiwalled carbon nanotubes [23]. Several important European Union‐funded projects have made significant contributions to the characterization of inorganic nanoparticles [3, 24, 25]. These include NanoDefine, NANoREG, and NanoValid under the Seventh Framework Program and Prosafe under the Horizon 2020 program [26, 27]. These projects focused on the development of common methods and frameworks for the physiochemical characterization and regulatory risk assessment of inorganic nanomaterials. The distinct elemental composition, crystalline structure, structural rigidity, high refractive index, and mass contrast of inorganic nanomaterials make them relatively easy to distinguish from the background medium. Therefore, their particle size characterization can be achieved using conventional analytical methods such as electron microscopy, field flow fractionation, light scattering, particle tracking analysis (PTA), and through advanced techniques such as single particle operation mode inductively coupled plasma mass spectroscopy [28, 29]. Organic nanomaterials that are entirely composed of soft carbon‐based polymers have low refractive index, low mass contrast, and weak light scattering properties compared to inorganic nanomaterials [30]. This is the reason why PNCs are not included within the scope of the OECD 125 test guidelines, and no specific recommendations have yet been provided for characterizing the particle size of PNCs.

Different analytical techniques measure particle size distribution based on distinct measurement principles (e.g., number‐based or intensity‐based), which results in discrepancies in measurement, even for a relatively simple polystyrene PNC standard and complex laboratory‐scale poly(ethylene oxide)‐block‐poly(caprolactone) PNCs [31, 32, 33, 34]. Commercial PNC suspensions synthesized on a large scale contain ASs and proprietary dispersants, and they are thus more complex than the polystyrene PNC standard. The applicability of different particle size techniques has not been systematically tested and critically assessed for complex commercial PNCs. Our study addresses this gap, which is essential to fill in order to advance the development and regulatory assessment of PNCs.

As a case study for this work, we selected a series of acrylic acid‐based PNCs, which are widely used for delivering ASs in agriculture, pharmaceuticals, and food applications owing to their hydrophilic and pH‐responsive properties [3, 35]. The commercially available PNCs were crosslinked and loaded with azoxystrobin (pesticide). The loaded AS refers to the AS associated with and on the surface of the PNCs, i.e., not only within the core of PNCs like a nanocapsule would be. Azoxystrobin was used as a model AS, commonly loaded into PNCs to improve the solubility and facilitate targeted delivery of the ASs. A non‐nano version (non‐crosslinked) of PNCs was included, with the aim to find nanospecific effects, following EFSA recommendations [1, 36]. Empty PNCs (without azoxystrobin) were also included to assess possible interferences due to the AS on size characterization. Laboratory‐scale PNCs synthesized in‐house were used to evaluate the impact of commercial dispersants on particle size distribution and to find the suitability of using laboratory‐scale PNCs as models for commercial‐scale PNCs in the regulatory risk assessment. A literature review on different PNC characterization techniques has highlighted the need for multiple complementary techniques for PNC size characterization [37]. Therefore, the size distribution of the five PNCs was characterized by cryo‐transmission electron microscopy (cryo‐TEM), DLS,PTA, and differential centrifugal sedimentation (DCS). The four analytical techniques were selected as they allow the characterization of the PNCs in their hydrated state, as drying can significantly affect the particle size, agglomeration, and surface characteristics of PNCs (Figure S1). The main objectives were to (i) assess the sensitivity, detection limits, and applicability of the techniques and (ii) to interpret discrepancies between the commercial product and the four PNC models. All characterization and data analysis were carried out following the OECD Guideline 125 [23] when possible (discussed in detail in Section 2).

## 2 Materials and Methods

### 2.1 PNC

Four types of commercial‐scale acrylic acid (AA, average molecular mass ~450 kDa)‐based PNCs were sourced from Vive Crop Protection Inc. (Toronto, ON, Canada): with and without crosslinking and with or without loaded AS (CL, NCL, CL‐AS, and NCL‐AS, respectively). Laboratory‐scale PNCs were synthesized in‐house following the procedure patented by Vive Crop Protection [38]. The synthesis involved the interaction between oppositely charged AA polymer chains (ca. 450 kDa, purchased from Sigma Corp.) and divalent metal counterions (M^2+^), resulting in condensed spherical structures (Figure 1A). The structural integrity of the PNCs was stabilized by UV crosslinking at 254 nm (Figure 1B). The counterions were removed from the PNCs using HCl and filtered through a dialysis membrane (10 kDa, Thermo Scientific, SnakeSkin dialysis tubing) before being lyophilized for long‐term storage (Figure 1C) [38]. Working suspensions were made from lyophilized stocks and resuspended in deionized water. These PNCs synthesized in‐house were compared with those synthesized at commercial scale in order to determine the effect of proprietary dispersants on size measurements and to assess whether laboratory‐scale PNCs are a suitable model when conducting regulatory risk assessment studies.

**FIGURE 1:**
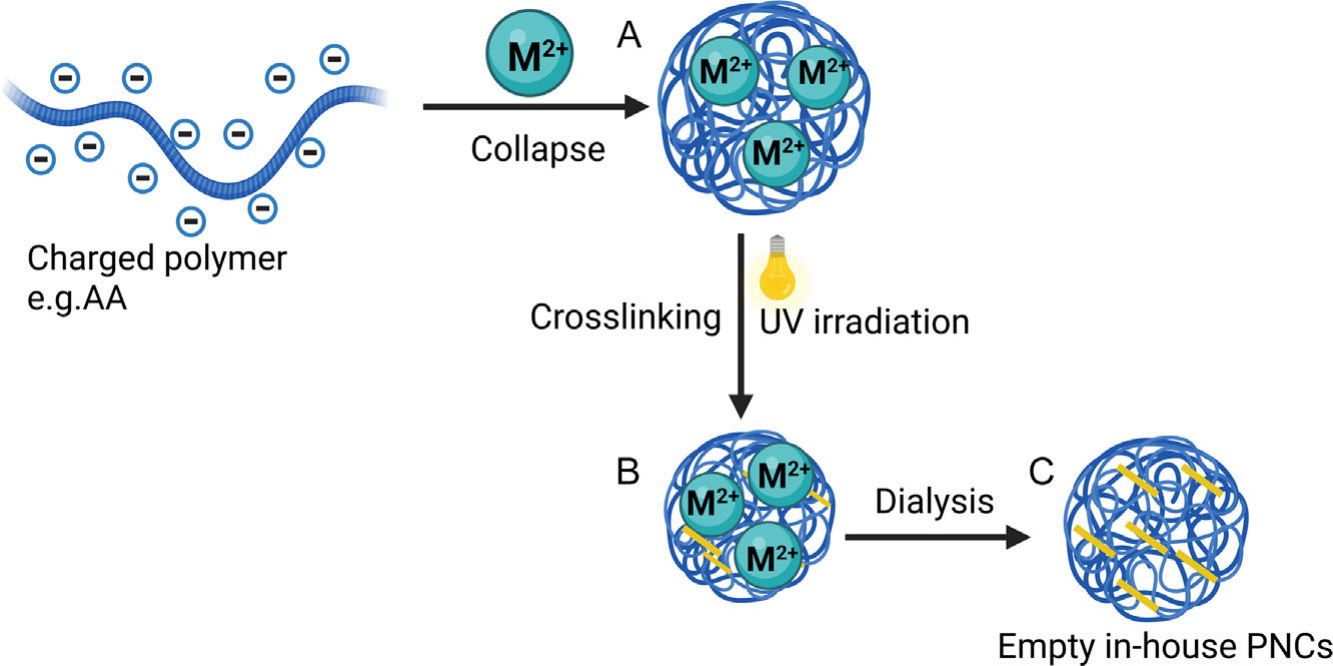
Schematic diagram of the synthesis of AA‐based PNCs: (A) the interaction between oppositely charged polymer chains and metal counter ions results in the collapse of the polymer chains, (B) stabilization by UV crosslinking at 254 nm, (C) removal of counterions by dialysis at a low pH of 1.5. Reprinted with permission from ref. [39] (American Chemical Society copyright 2026).

### 2.2 Cryo‐Transmission Electron Microscopy (cryo‐TEM)

A Tecnai FEG20 TEM microscope with a 200 kV electron beam energy was used for imaging. In bright‐field cryo‐TEM imaging, the empty and loaded PNCs were imaged in a frozen‐hydrated state on carbon‐coated micron‐hole grids with a diameter of 1.2 µm and a hole spacing of 1.3 µm on a copper square mesh (Protochips, USA). The glow discharge was applied to hydrophilize the grids, facilitating the optimal spread of the sample. Then, the grid was positively charged using 10 µL of 5 mM magnesium acetate solution to attach the negatively charged PNCs on the grid surface. The application of magnesium acetate modifies the surface properties of the carbon grid by introducing a positive charge, and this facilitates the spread of negatively charged empty and loaded PNCs on the positive carbon grid. However, PNCs observed within the holes and on the carbon grid exhibited similar morphologies (Figure 2A,B,D), suggesting that magnesium acetate did not induce changes in the particle morphology. Without magnesium acetate, the number of particles observed within the holes was insufficient to obtain the statistical size distribution further increase in concentration of particles was not possible as we have already used undiluted commercial formulation for the imaging. Therefore, in this particular case, magnesium acetate was introduced during method optimization to increase the number of particles across the grid. For comparison, conventional TEM imaging was also conducted using a thin continuous carbon grid. As small PNCs <100 nm were not observed in conventional TEM imaging (Figure S2), cryo‐TEM was selected for further analysis.

**FIGURE 2:**
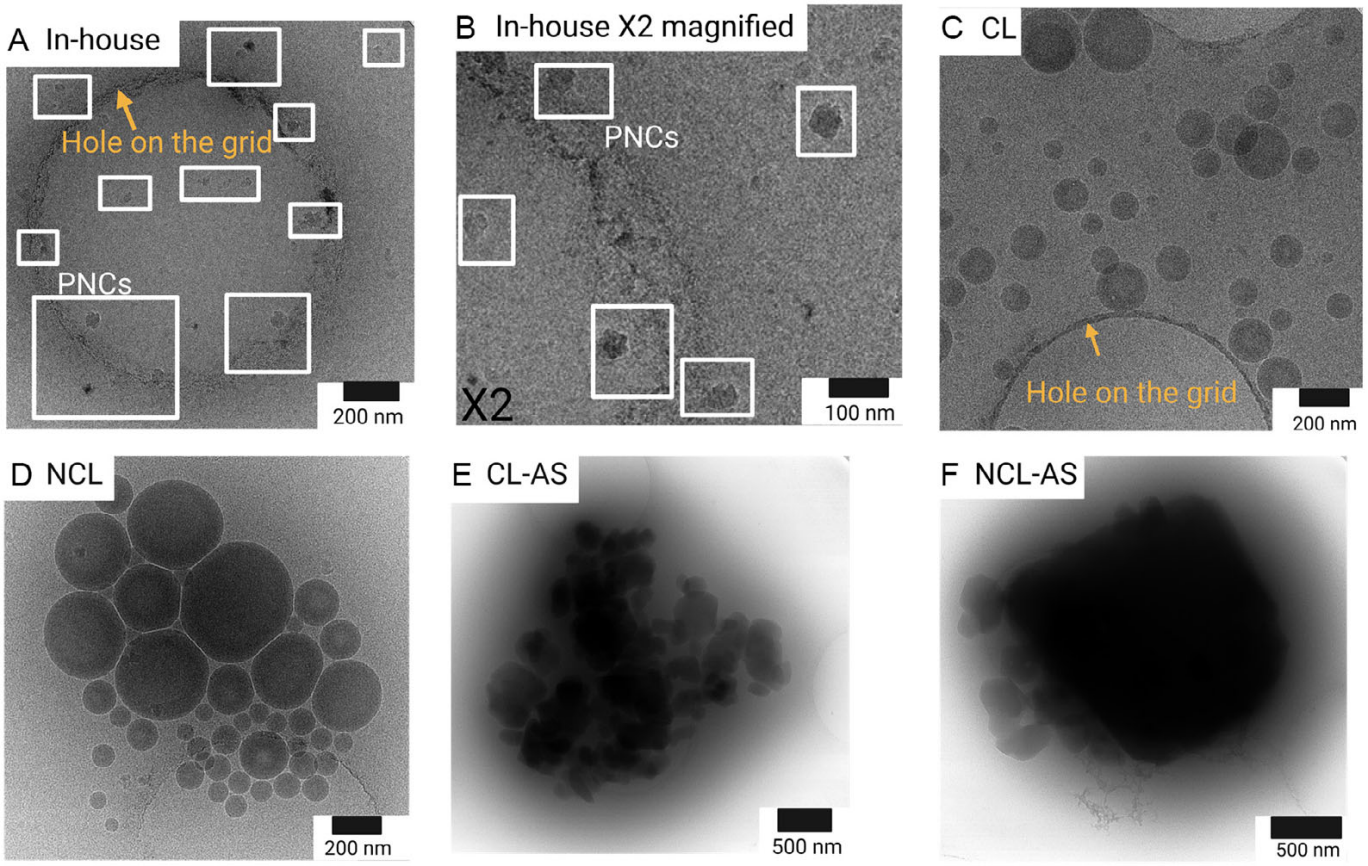
Cryo‐TEM images of (A) in‐house, (B) a X2 magnified view of in‐house shown in (A), (C) crosslinked polymer nanocarriers (CL), (D) noncrosslinked polymer nanocarriers (NCL), (E) CL PNCs loaded with active substance (CL‐AS), and (F) NCL PNCs loaded with AS (NCL‐AS).

However, it is important to consider that soft PNCs attached onto the carbon grid prior to plunge‐freezing may undergo flattening, particularly for larger particles (>200 nm, Figure S1C). The excess magnesium acetate on the grid was removed by blotting using filter paper, leaving behind a thin film of magnesium acetate on the grid. After 10 min of sonication, 10 µL of the non‐diluted empty PNC sample was deposited on the grid inside a humidity chamber with a relative humidity maintained at 100% at 4 °C to prevent the particles from drying (Vitrobot, Thermo Fisher). The similar morphologies of the particles within and outside holes (Figure 2A,B,D) further support that drying of particles outside the holes was very limited. For AC‐loaded PNCs, 10 µL of diluted PNCs at an overall mass concentration of 200 mg/L in deionized water was used. The excess sample was blotted using filter paper within the Vitrobot. Then, the sample was plunge freezed in liquid ethane (−183 °C). A high cooling rate was used to vitrify the sample, without the formation of ice crystals (cubic or hexagonal) and freeze‐drying artifacts (Figure S3A–C) [40]. The thickness of the embedded ice film depends on several factors, such as the viscosity of the medium and blotting time. Therefore, soft PNCs with a larger diameter tend to flatten due to capillary effects and surface forces (Figure S1C). In this study, the flattening was more evident for PNCs >200 nm. Therefore, the cryo‐TEM size measurements of large PNCs should be interpreted with caution. After freezing, the samples were mounted on a cryo‐specimen holder at −175 and −180 °C. The images were recorded digitally using a 16 M pixel TVIPS XF416 ES camera (Tietz video and image processing systems GmbH, Gilching, Germany) under low‐dose conditions to limit the detrimental effects of electron beam damage on PNCs (Figure S3D,E). The size distribution from cryo‐TEM was determined by manually counting nonoverlapping, clearly distinguishable particles (≥300 for monodisperse and ≥700 for polydisperse suspensions), in accordance with the OECD Test Guideline 125 [23]. In this study, PNCs were polydisperse; therefore, a total of 750 particles were analyzed, from 54 images for the NCL PNCs, 37 images for the CL PNCs, and 101 images for the in‐house PNCs. The diameter of these particles in the cryo‐TEM images was measured using ImageJ (image processing software) version 1.54. The particle size distribution is reported using geometric parameters based on a log‐normal distribution model [23].

### 2.3 DLS

All DLS measurements were performed at 25 ± 1 °C, with a Malvern Zetasizer Nano instrument equipped with a Helium‐Neon laser and a backscatter detector at an angle of 173°. Three replicates of the PNC suspension diluted in deionized water were analyzed at an overall mass concentration of 200–300 mg/L. The overall PNC mass concentration in commercial PNCs was determined by freeze‐drying a known volume of PNC suspension. Determining the concentration in particles per volume (mL) was challenging due to the PDI of the PNCs. The samples were sonicated for 10 min before the DLS analysis under the following conditions: refractive index 1.53 [41], equilibration time 2 s, 11 runs, and 3 measurements per replicate. Correlograms were analyzed using a multiple narrow peak algorithm to obtain intensity‐based average hydrodynamic diameter measurements [5, 42]. Following the OECD 125 guideline [23], the reproducible particle size measurements at identical dispersing conditions were used for the data analysis.

### 2.4 PTA

PTA was conducted in triplicate with a Zetaview PTA (Particle Metrix, GmbH, Germany) instrument, equipped with a 488 nm laser. The samples were analyzed after 10 min of sonication, with five different measurements per replicate, following the OECD test guideline 125 [23]. The PTA was carried out at relatively low concentrations compared to DLS (10–35 mg/L for empty PNCs and 1 mg/L for AS‐loaded PNCs). The sample concentration used in PTA is further discussed in detail in the results and discussion section. The PTA was conducted under the following conditions: cell temperature 25 °C, sensitivity 80, shutter 100, maximum Area 1000, minimum Area 10, and minimum Brightness 20. PTA Software v 8.06.01 was used for data capture and analysis. Similar to DLS, reproducibility criteria under identical dispersing conditions were applied for PTA [23].

### 2.5 DCS

DCS was conducted in triplicate using a CPS 24 000‐disc centrifuge (CPS Instruments Inc., Stuart, Florida, USA). The instrument operated at 24 000 rpm. The density gradient required for analysis was obtained by sequentially injecting two sucrose solutions with different concentrations at the center of the disc. During the disk rotation, the two solutions partially mix at the disc center, resulting in a sucrose concentration gradient across the disc with the concentration of the first injected sucrose solution at the outer side of the disc and the second one at the center of the disc. For particles with low density, i.e. 1.04 g/mL (namely CL, NCL, and in‐house PNCs) 2% and 8% sucrose solutions were used. For AS‐loaded PNCs (i.e., CL‐AS and NCL‐AS) that have a high particle density (average of 1.14 g/mL), 4% and 12% sucrose solutions were used. The samples were prepared in deionized water at an overall mass concentration of 2 g/L and sonicated for 10 min to ensure a uniform dispersion of the PNCs. After sonication, 0.1 mL of the suspension was injected into the instrument for analysis. The results obtained were analyzed in terms of both mass and number distribution. Similar to DLS and PTA, the OECD reproducibility criteria were also applied [23].

### 2.6 Statistical Analysis

Statistical analysis of the data obtained was performed using GraphPad Prism software (version 10.6.1). Tukey's two‐way ANOVA with multiple comparisons tests was conducted on the particle size data obtained from different techniques. A 95% confidence interval was applied to each comparison. For the statistical tests, a significance threshold of 0.05 was used, with all significant differences discussed below returned *p*‐value <0.0001.

## 3 Results and Discussion

Four different particle size characterization techniques were systematically applied to five different PNCs, all polydisperse spherical particles. The noncrosslinked particles (NCL) were included to assess the suitability of the techniques to characterize non‐nano PNCs and distinguish them from crosslinked nanocarriers (CLs). The in‐house PNCs were used to evaluate the impact of commercial dispersants on particle size distribution and to assess the suitability of using laboratory‐scale PNCs as models for commercial PNCs with the aim of supporting regulatory risk assessment. The AS‐loaded PNCs (CL‐AS and NCL‐AS) were compared with empty PNCs (CL and NCL) to assess how the presence of AS influences size measurement and to evaluate whether the size characterization data from empty PNCs can accurately represent the AS‐loaded PNCs (read across). The crosslinking creates smaller and more compact PNCs than the noncrosslinked versions. In this study, the lab‐scale synthesis conditions are expected to provide more control over the crosslinking compared to the commercial‐scale conditions; therefore, we expected the particle size to be in the order NCL > CL > in‐house. The following sections discuss the applicability of each technique and the discrepancies observed across different techniques and PNC suspensions.

### 3.1 Cryo‐TEM Analysis

The PNCs used in this study were soft, and they tend to deform during the conventional drying, which was confirmed using dry‐state SEM imaging (Figure S1). Therefore, in cryo‐TEM analysis, the PNCs are imaged in a vitrified, hydrated state to minimize the detrimental effect on PNCs, from melting and shrinkage caused by drying under high‐vacuum electron beam conditions. For all the different PNCs, the size distribution was determined by manually selecting nonoverlapping particles from the cryo‐TEM images. This is because the NCL PNCs exhibited some agglomeration, and large PNCs from CL and NCL PNCs were quickly damaged by the electron beam (Figure S3D,E). Therefore, the damaged and agglomerated particles were excluded, and well‐separated particles were manually selected for analysis. It was challenging to observe clear and well‐defined boundaries for some of the very small in‐house PNCs, less than 50 nm, due to the low electron density and the minimal sample thickness at the edges, resulting in weak contrast (Figure 2A,B). Furthermore, high‐magnification imaging was not possible due to the low electron beam tolerance of the in‐house PNCs. As expected, crosslinking resulted in small, compact particles of CL PNCs (Figures 2C and 3A) as compared to the larger NCL PNCs (Figures 2D and 3B). Comparing the in‐house PNCs with CL, other than the presence of comparatively smaller in‐house PNCs, no significant influence on particle size measurements was observed from the additional dispersants in commercial‐scale PNCs (Figure 2A,C). The agglomeration induced by AS made imaging them challenging, as evidenced by dark, nonspherical particles in the cryo‐TEM images (Figure 2E,F). The OECD criteria, which consider only no‐overlapping well‐defined particles, were not applicable to AS‐loaded PNCs, and therefore, it was not possible to determine the particle size of AS‐loaded PNCs using this technique.

**FIGURE 3:**
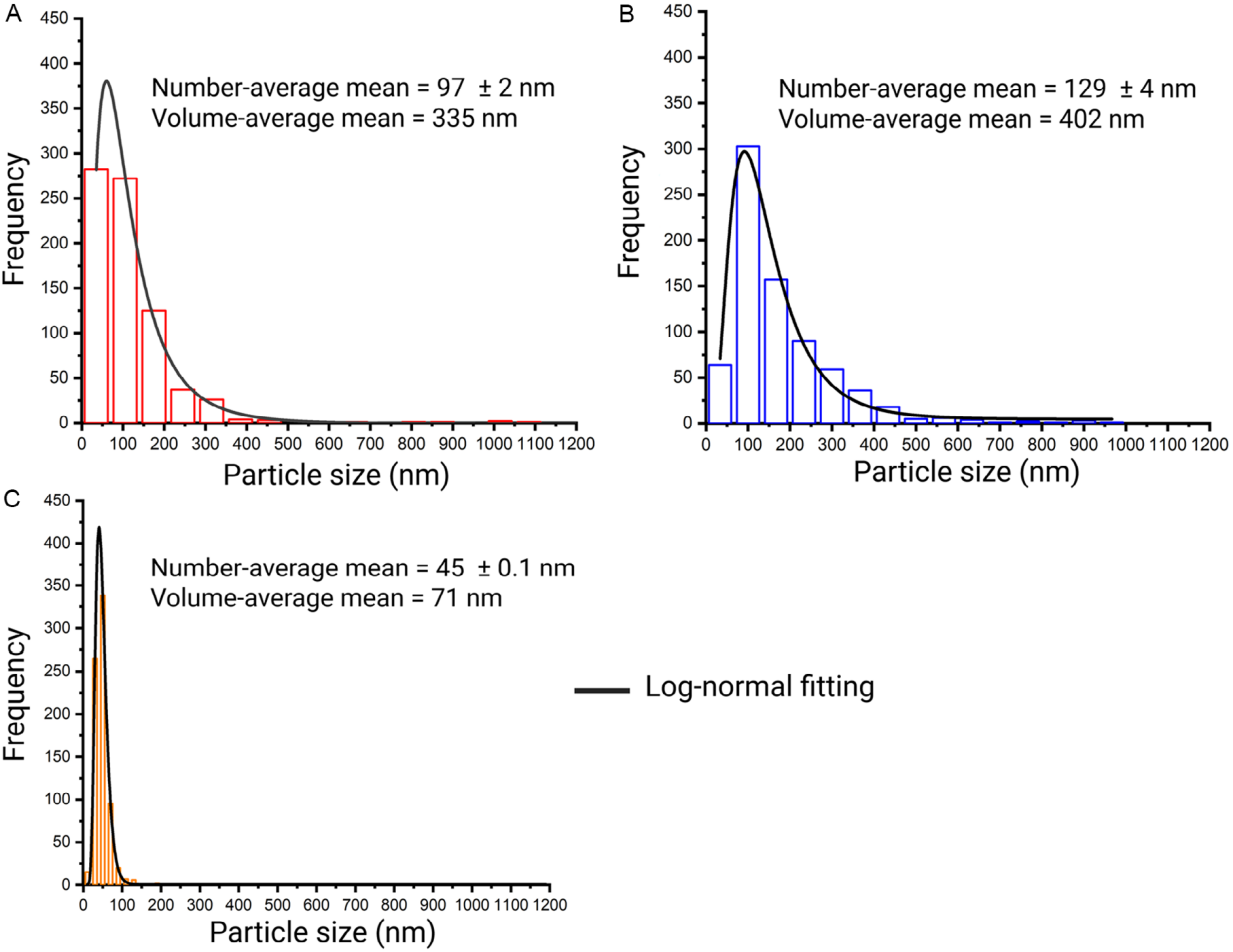
Log‐normal size distribution of (A) CL, (B) NCL, and (C) in‐house synthesized PNCs. The log‐normal fit is applied to the Feret diameter measured from the cryo‐TEM images. The number‐average mean represents the fitted geometric mean ± standard error of the fitted parameter.

The log‐normal fit was applied according to OECD Test guideline 125 [23] and literature [43], for reporting the particle size distribution of polydisperse nanomaterials that contain agglomerates or aggregates (Table S1). The CL PNCs had a broader size distribution compared to the in‐house PNCs, due to differences in the experiment setup or conditions used in the commercial‐scale synthesis (Figure 3A,C). A recent study reported similar findings that lipid nanomaterials produced at a commercial scale yielded different physical properties, despite using the same bench‐scale synthesis protocol, due to the difference in the mixing equipment used for the commercial‐scale synthesis [44]. Similar findings were also observed in our study. The laboratory‐scale in‐house PNCs were smaller (ca. 50 nm) than the commercial‐scale PNCs (ca. 100 nm). Therefore, laboratory‐scale models may not be a suitable substitute for commercial products in regulatory risk assessment studies.

However, it is challenging to achieve well‐defined boundaries for very small in‐house PNCs (Figure 2A,B) due to the weak contrast produced by the less electron‐dense thin edges. It is important to note that image defocus creates Fresnel fringes (halo rings around particles) at the particle boundaries, increasing the apparent size distribution of small in‐house PNCs. In addition, the flattening of large PNCs (>200 nm, as discussed in Section 2.2) may also affect cryo‐TEM size measurements. Therefore, for polydisperse commercial PNCs, the cryo‐TEM size distribution should be interpreted while considering these limitations.

Interestingly, some particles in the images of commercial PNCs exhibited a vesicle‐like morphology (Figure 2C,D). Vesicles were not expected given the patented synthesis protocol for the commercial PNCs [38]. Noting that laboratory‐scale PNCs did not exhibit a vesicle‐like morphology (Figure 2A,B) and that the commercial formulation includes undisclosed formulants that may appear as particles in TEM images, the identity of the vesicle‐like particles would need to be assessed with, e.g. small‐angle X‐ray scattering (SAXS) was performed to confirm that they are indeed PNCs. In addition, cryo‐TEM distinguishes empty CL PNCs from NCL PNCs (Figures 2C,D and 3A,B). However, the agglomerated PNCs are not represented in the size analysis, as they were excluded to meet the OECD microscopy guidelines (as discussed in Section 2.2). This challenges the realistic representation of non‐nano PNCs and the cryo‐TEM data comparison with other techniques (DLS, PTA, and DCS) that process all data together without excluding any sample fraction. This challenges the applicability of the OECD guidelines for microscopy to AS‐loaded PNCs due to the agglomeration induced by the loaded ASs. Contrast enhancements were attempted on the images of the AS‐loaded PNCs (Figure S4). Although this improved the visibility of the particles, the boundaries of particles remain insufficiently sharp to derive a particle size distribution. However, it is important to note that there are different approaches to count particles. For instance, each aggregate and agglomerate can be counted as one particle or alternatively, individual particles, touching particles, and integral components in agglomerates can be counted as individual particles; different ways of counting particles directly affect the determination of the size distribution [2, 45]. Our results also suggest that read across between empty and loaded PNCs may not be a valid approach.

### 3.2 DLS Analysis

DLS analysis is a convenient method commonly used to indirectly measure the hydrodynamic diameter of spherical PNCs. In DLS, the translational diffusion coefficient of particles is calculated from the time‐dependent fluctuations in scattered light and converted to the hydrodynamic diameter using the Stokes–Einstein equation (Equation (1)) [46]. Whereas, PDI is the dimensionless measure of the width of the particle size distribution. PDI is calculated from the cumulant analysis of the intensity autocorrelation, as the ratio of the second cumulant to the square of the first cumulant [47].

(1) $$D_{h}=\frac{kT}{3\Pi \eta D_{t}}$$

Where *D*_h_ = hydrodynamic diameter, *k* = Boltzmann's constant, *T* = temperature, *η* = solution viscosity, and *D*_t_ = translational diffusion coefficient (Brownian motion velocity) [46].

DLS is not applicable to polydisperse suspensions because large particles mask the smaller particles from detection. In Rayleigh scattering, the scattering intensity depends on the 6^th^ power of particle size, and the intensity of light scattered by a small proportion of large particles or agglomerates dominates the size measurement data in DLS [48]. The masking by large particles was further confirmed by the tailing observed in the average particle size measurements of all PNC samples, except for the in‐house PNCs (Figure S5, dotted red box). The tailings were due to the significant contribution of few large particles or agglomerates to the overall scattering [49]. Therefore, it was not possible to distinguish nano‐PNCs from non‐nano‐PNCs using DLS. For all the PNCs, DLS results indicated average hydrodynamic particle sizes of >300 nm (Figure 4A), which is inconsistent with measurements from cryo‐TEM images, where all PNCs were found to be around 100 nm and the in‐house PNCs were around 50 nm (Figures 2 and 3). This discrepancy is not due to the hydration shell around PNCs in solution (a common attribution factor mentioned in studies) but is due to the presence of polydisperse and nonspherical PNCs. The AS‐induced agglomeration resulted in irregular clusters (Figure 2E,F), making it difficult to recognize the translational diffusion of these nonspherical particles using the simple geometric model used in the DLS algorithm [34].

**FIGURE 4:**
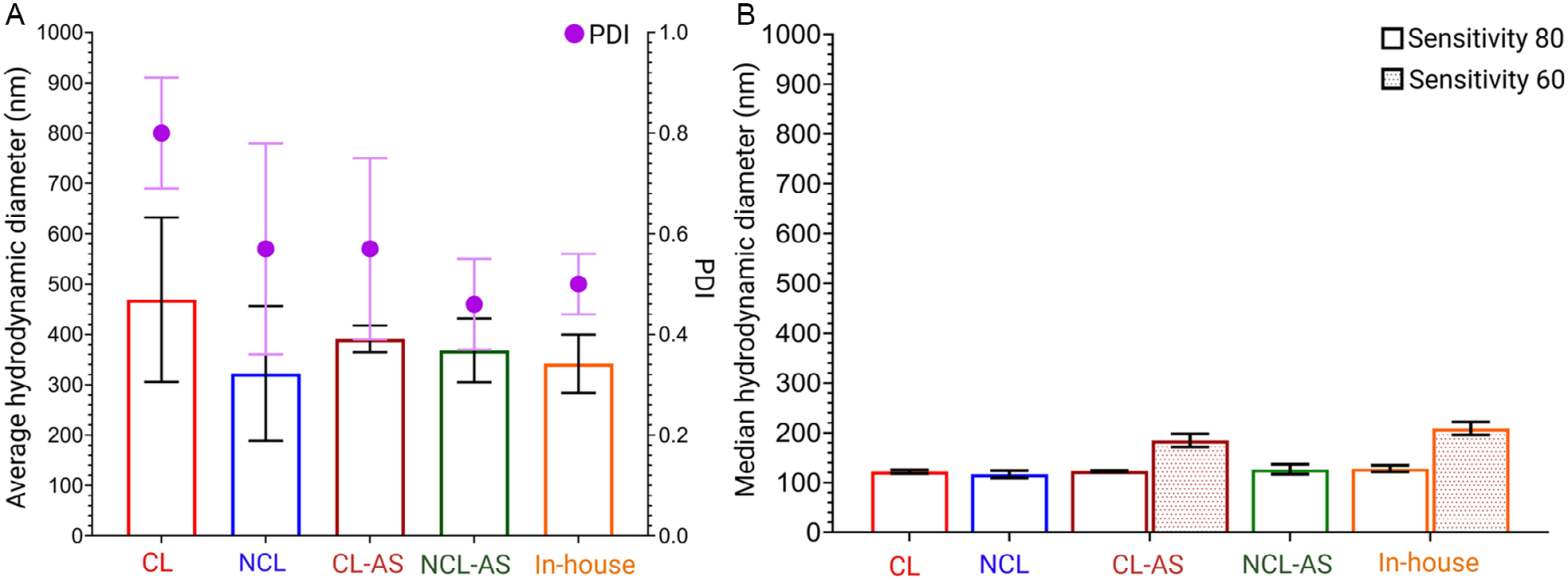
The average and median hydrodynamic particle size measurements obtained from PTA of different types of PNCs: crosslinked (CL), noncrosslinked (NCL), AS‐loaded crosslinked (CL‐AS), AS‐loaded noncrosslinked (NCL‐AS), and laboratory‐scale in‐house synthesized (in‐house). For the DLS analysis (A), the high polydispersity (right axis, purple circles) indicates that DLS is not a suitable analytical approach (also shown by the large error bars from triplicates. For PTA (B), the median hydrodynamic diameter is shown at two different preacquisition sensitivity values, 80 (unshaded) and 60 (shaded).

As a result, the DLS measurements are not applicable to nonspherical particles, and the particle size data obtained from irregular nonspherical AS‐loaded PNC clusters are misleading [34]. The average polydispersity (PDI) was high for all five different PNCs (ranging 0.5–0.8, Figure 4A), confirming the broad size distribution and PDI of the PNC suspensions. In addition, OECD reproducibility criteria [23] were not achieved in DLS (Figure 4A).

In summary, DLS is commonly used for characterizing PNCs, assuming that the particles are spherical and monodisperse. However, these assumptions do not always hold. Based on our cryo‐TEM analysis, the AS‐loaded PNCs can deviate from the spherical geometry observed in the corresponding empty PNCs. Therefore, the spherical geometry of empty PNCs is insufficient to justify the use of DLS for loaded PNCs, which can lead to inaccurate conclusions about the properties of PNCs. Therefore, microscopic imaging of AS‐loaded PNCs is recommended to confirm the particle morphology and the suitability of DLS. However, DLS is suitable for comparing particles of a similar nature. DLS is also used in the literature to characterize the particle size distribution of polydisperse PNCs when the size populations are sufficiently separated, and it is challenging otherwise [33]. While DLS is useful for comparing the relative agglomeration of samples over time, using DLS as a method to determine the particle size of PNCs is not recommended unless they are demonstrated to be monodisperse and spherical.

### 3.3 PTA

PTA tracks the Brownian motion of individual spherical PNCs by a video camera to determine the translational diffusion coefficient of PNCs. The translational diffusion coefficient is applied to the Stokes–Einstein equation (Equation (1)) to calculate the hydrodynamic diameter of the PNCs [50]. Unlike DLS, PTA does not report average particle size from ensemble measurement [51]. Due to the single‐particle tracking approach, PTA is less susceptible to the influence of large particles in solution, and it is considered suitable for polydisperse samples even in the presence of agglomerates [49].

PTA was conducted at different concentrations for empty PNCs and AS‐loaded PNCs. This is because the presence of ASs made the PNC suspension complex and challenging to track the individual trajectory of PNCs. To facilitate individual tracking, the AS‐loaded PNCs had to be diluted to a very low concentration (1 mg/L) compared to empty PNCs (10–35 mg/L). It was not possible to conduct PTA at an identical concentration for empty and loaded PNCs, which challenges the comparisons.

PTA met the repeatability criteria of the OECD 125 guideline, and no significant influence from the additional dispersant molecules was observed in PTA (all PNCs returned similar sizes, Figure 4B). Maintaining identical preacquisition parameters, such as sensitivity, was essential in obtaining these reproducible results. To evaluate the effect of sensitivity on the particle size determination, PTA was conducted at two preacquisition sensitivity settings (80 and 60) on AS‐loaded and empty laboratory‐scale PNCs (CL‐AS and in‐house PNCs). The CL‐AS and in‐house PNCs represent the two different types (loaded and empty) and size classes of all the PNC samples under investigation (Figures 4B and S6–S13). The PTA sensitivity values range from 0 to 100 and can be optimized based on the scattering intensity of the samples and the complexity of the background solution of interest. Increasing sensitivity values amplifies the scattering signal, allowing more small particles of low refractive index to be detected. However, this can saturate the PTA frame with too many bright features arising from large particles that scatter more light, resulting in the exclusion of large particles of PNCs in PTA at high sensitivity; this is an important result for PNCs (Figure 4B) [51]. As a result, no evidence of large particles or AC induced agglomeration was found, and nano‐ and non‐nano PNCs yield similar median particle size measurements in PTA (Figure 4B).

Preacquisition sensitivity parameters affect the particle size determination of both inorganic and organic nanomaterials, but this effect is more significant for organic nanomaterials, which typically have a low refractive index. For instance, for a silica–gold core–shell nanoparticle suspension (refractive index, 2.25) [52], using a medium‐sensitivity parameter, nanoparticles of around 10 nm can be detected without saturating the detector [50]. Whereas, for polydisperse PNCs, a high sensitivity setting is required due to their low refractive index (1.53) [41] and this can saturate the detector with the excessive bright signals from large particles. For loaded and empty PNCs, larger median hydrodynamic particle sizes were derived from measurements at lower sensitivity (e.g., for CL‐AS, 185 ± 13 and 123 ± 2 nm at 60 and 80 sensitivities, respectively). At low sensitivity, less particles were present in the frame (e.g., for CL‐AS, an average of 50 and 100 particles per frame at 60 and 80 sensitivities (Figure S12A,B). Similar results were also obtained for in‐house PNCs (209 ± 13 and 128 ± 7 nm at sensitivity 60 and 80, respectively) (Figure S13). It was thus not possible to select a single optimum sensitivity setting for the entire range of PNCs. For instance, reducing the sensitivity setting to 60 did not allow the detection of approximately half of the small 100 nm PNCs (Figure S12), resulting in an underestimation of the number of small PNCs. Filtering out large particles is an option if the aim is to verify the presence of small PNCs in a polydisperse PNC suspension. However, the PNCs below the detection threshold may still remain undetected due to weak light scattering properties, e.g., small in‐house PNCs <50 nm were not detectable by PTA (Figure 4B).

Overall, while PTA is generally recommended for characterizing polydisperse nanomaterials, it was not well adapted to characterize our polydisperse PNCs. PTA results for organic nanomaterials should be carefully interpreted, taking into account the nanomaterial‐specific properties (e.g., refractive index) and the analytical limitations associated with the PTA instrument.

### 3.4 DCS Analysis

The DCS analysis determines the hydrodynamic size of the spherical PNCs based on the sedimentation velocity [53]. The PNCs are injected into the center of a spinning DCS disc and spread over the surface of the fluid, characterized by a gradient in density and viscosity along the radial direction, obtained by injecting sucrose solutions at different concentrations before the measurement is performed. Based on their density and size, the PNCs sediment radially at different velocities at the outer edge of the centrifugation disc. The turbidity detector at the outer edge of the disc records the change in scattered light from a monochromatic light source as a function of time, and the intensity‐based particle size is calculated using the Stokes law [53]. DCS is generally considered suitable for polydisperse PNCs as agglomerates can be detected as a distinct subpopulation among individual particles (Figures S14–S18) [53].

The DCS was able to detect large particles or agglomerates present in NCL PNCs and AC‐loaded PNCs (Figures S15–S17). The significant amount of agglomeration found in AC‐loaded PNCs was clearly identified by DCS analysis (Figures S16 and S17). DCS analysis provided an overview of the size distribution in a single instrument run; there was no role of preacquisition parameters here, and millions of particles were analyzed within minutes, resulting in statistically robust data [54]. Conversely, the detection limit of DCS depends on the maximum rotational speed achieved by the DCS [55]. The higher the rotational speed, the greater the centrifugal force, and the faster the path of the particles to the detector, facilitating the sedimentation of small PNCs. However, the maximum speed achieved by DCS (24 000 rpm) limits the detection of the PNCs to 50 nm (Figure S18). Smaller particles would require a travel time too long (several hours) to maintain a stable sucrose gradient (and therefore to consider the measurement reliable at long times). Due to the difference in sedimentation properties, DCS was able to distinguish nanoempty and loaded PNCs from their corresponding non‐nano versions (Figure 5). Compared to commercial models, smaller in‐house PNCs were detected using DCS; other than this difference in particle size, no significant impact of the commercial dispersants on the particle size was observed (Figures S14 and S18).

**FIGURE 5:**
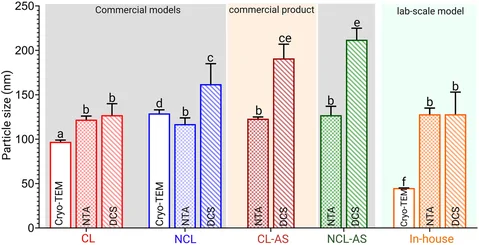
The comparison between particle size measurement of different types of PNCs: crosslinked (CL), non‐crosslinked (NCL), AS‐loaded crosslinked (CL‐AS), AS‐loaded non‐crosslinked (NCL‐AS), and laboratory‐scale in‐house synthesized (in‐house) obtained from different size estimation techniques such as cryo‐transmission electron microscopy (cryo‐TEM, log‐normal distribution), particle tracking analysis (PTA, median hydrodynamic diameter), differential‐centrifugal sedimentation analysis (DCS, cumulative number distribution). Datasets that share a common letter were statistically similar, based on Tukey's test.

Overall, in the DCS, the sedimentation property plays a crucial role in separating polydisperse PNCs of different particle sizes. DCS was applicable for detecting the particle size of empty and loaded PNCs greater than 50 nm. However, the particle size measurements for AS‐loaded PNCs need to be interpreted carefully, considering their nonspherical geometry and the actual density of the individual particles.

### 3.5 Comparison Between Techniques

Some differences were expected between the results from DLS, cryo‐TEM, PTA, and DCS due to the differences in the principles behind each technique. Such discrepancies have been extensively reported for inorganic NPs. For instance, for a given type of gold NPs, PTA and DLS tended to generate larger sizes (22 and 57 nm, respectively), compared to DCS, SEM, and TEM (11, 13, and 15 nm, respectively) [56]. The unsuitability of DLS to measure polydisperse samples applies to both inorganic NPs and PNCs. Large particles result in intense scattered light as compared to small particles, contributing to an overestimation of the hydrodynamic size distribution, as previously shown, for, e.g., inorganic silica nanoparticles [57]. The same was observed for our PNCs: values obtained by cryo‐TEM were significantly smaller than those obtained by PTA and DCS (*p *< 0.0001). This difference is due to the number‐based particle size distribution obtained from cryo‐TEM, which underestimates the contribution of the largest particles. In order to consider the contribution from large particles, the number‐average mean can be converted to the volume‐average mean based on Equation (2) (Figure 3) [33, 58]. The ratio between the volume‐average diameter and the number‐average diameter represents the PDI from the cryo‐TEM distribution, which is greater than 1 for CL, NCL, and in‐house PNCs due to the asymmetry of the log‐normal distribution. In addition, the size measurements from PTA and DLS are intensity‐based average mean that measure the hydrodynamic diameter, which includes the hydration layer around PNCs and is not accounted for in cryo‐TEM. Although the number‐average distribution was converted to volume‐average particle size distribution, the difference was particularly striking for the smallest PNCs (i.e., in‐house), confirming the inability of NTA and DCS to detect small PNCs due to their low refractive index and low density. Direct numerical comparison between cryo‐TEM, DLS, and PTA should be interpreted with caution. An overview of the advantages and challenges associated with each technique in characterizing polydisperse empty and loaded PNCs is summarized in Table 1.

**TABLE 1:** Overview of the advantages and challenges associated with particle size characterization techniques for polydisperse empty and loaded PNCs.

Advantages	Challenges
Cryo‐TEM	
• Only technique that could detect empty and loaded PNCs <50 nm	• Low contrast and challenging to obtain well‐defined boundaries for PNCs <50 nm and PNC‐ASs
• Distinguish empty PNCs from loaded PNCs and crosslinked from noncrosslinked PNCs	• AS‐induced agglomeration limits its application in finding the size distribution of loaded PNCs
• Identify agglomerates from individual particles	• For polydisperse empty and loaded PNCs, large particles >200 nm get flattened and the appearance of Fresnel fringes on particles <50 nm affects the size distribution data
	• Size distribution is influenced by the application of different counting rules
	• Excludes a definite fraction of PNCs and PNC‐ASs to meet the particle counting guidelines
	• Limited accessibility
DLS	
• Convenient and fast method	• The assumptions of spherical geometry are not always applicable, e.g., PNC‐AS
• Suitable for the quick comparison of PNCs of a similar nature	• More susceptible to the influence of large particles
• Includes entire particle population	• Not suitable for polydisperse empty and loaded PNCs
	• Challenging to distinguish empty PNCs from loaded PNCs and crosslinked from noncrosslinked PNCs
PTA	
• Less susceptible to the influence of large particles	• Size distribution dependent on preacquisition parameters
• Individual tracking of particles	• Challenging to characterize the entire particle size range of polydisperse empty and loaded PNCs under a single preacquisition setting
	• A definite fraction of the particle population is excluded from data analysis due to the preacquisition settings
	• Challenging to distinguish empty from loaded PNCs
	• Not adapted for detecting polydisperse empty and loaded PNCs with low RI
DCS	
• Independent of preacquisition parameters	• Detection limit of <50 nm for empty and loaded PNCs
• Large particles can be detected as a distinct subpopulation	• The assumption of spherical geometry does not always hold for loaded PNCs; hence, microscopic verification is recommended
• Distinguish empty PNCs from loaded PNCs and crosslinked from noncrosslinked	
• Identify agglomerates from individual PNCs	
• Millions of PNCs and PNC‐ASs are analyzed within minutes, resulting in statistically robust data

(2) $$\mathrm{Volume}\;\mathrm{average}\;\mathrm{particle}\;\mathrm{size}=\frac{\Sigma_{n_{i}}D^{3}}{\Sigma_{n_{i}}D^{2}}$$

Where *n*_*i*_ represents the number of particles with diameter *D*_*i.*_

Inorganic nanomaterials that have high density and refractive index can be relatively well characterized by a single method, such as cryo‐TEM, PTA, or DCS. Our results show that a multitechnique approach is essential for empty and loaded PNCs, especially when the objective is to characterize unknown commercial suspensions that are likely to be polydisperse. While DCS could deal with PDI, cryo‐TEM was the only technique that could detect the presence of PNCs <50 nm. Smaller particles are of particular interest for risk assessment because they may be able to cross biological barriers [59]. It is thus essential to apply an analytical strategy that covers the entire size distribution of PNCs.

### 3.6 Comparison Between PNCs

Differences were expected between the laboratory‐scale, in‐house, and commercial‐scale crosslinked PNCs (CL, CL‐AS) (Figure 5), as previously reported for lipid nanoparticles [44]. The synthesis conditions, such as stirring, mixing, and UV exposure, differed due to the differences in the equipment and chemical grades used at the laboratory and commercial scales, which affected the size distribution. This confirms that lab‐scale PNCs are not suitable models for characterizing commercial PNC products. The presence of loaded ASs also significantly influenced the particle size and shape of PNCs. According to the DCS, the CL and NCL PNCs significantly differed from the CL‐AS and NCL‐AS PNCs (Figure 5). Therefore, we highly recommend conducting characterization, exposure, and hazard assessments on the commercial final product (CL‐AS) in order to avoid misleading results from inadequate model PNCs.

## 4 Recommendations and Conclusion

Our attempts to characterize the size of five empty and loaded PNCs using four different techniques (i.e., cryo‐TEM, PTA, DLS, and DCS) lead to the following recommendations.

### 4.1 Sample Preparation

Unlike many “hard” inorganic nanoparticles, the particle size, agglomeration, and surface characteristics of soft PNCs and PNC‐ASs vary significantly between the dry and hydrated states. PNC characterization in the hydrated state should thus be preferred if it is representative of the use scenario.

Sample preparation should be carefully considered and reported fully to ensure the reproducibility of particle size measurements. For instance, for cryo‐TEM, maintaining a thin layer of vitreous ice is important for PNC imaging as increased ice thickness significantly diminishes the contrast of the PNCs.

In PTA and DCS, a high concentration of PNCs can improve the signal and may be needed in order to detect PNCs. However, it can also induce agglomeration, which may not be representative of use scenarios occurring at lower concentrations. For PTA, working at a sample‐specific optimal concentration is also essential. Concentration effects should thus be carefully considered when interpreting the results and when comparing different PNCs.

### 4.2 Preacquisition Parameter

We recommend that PTA size distributions should be reported along with the preacquisition parameters, such as sensitivity, as those can significantly affect the reproducibility of the analysis. For an unknown commercial PNC suspension, the expected particle size range is uncertain. Hence, a preliminary analysis using electron microscopy is recommended to optimize the sensitivity settings specific to the PNC or PNC‐ASs samples prior to PTA. The polydisperse PNCs are very challenging to analyze using a single‐sensitivity setting in PTA. If considering PTA for polydisperse PNCs and/or PNC‐ASs, characterization with at least two different sensitivity conditions is recommended.

### 4.3 Sample Geometry

The DLS, PTA, and DCS analyses assume a spherical particle geometry, which may not always be true, as shown here for the AS‐loaded PNCs. Verifying assumptions by, e.g. microscopy will help improve the accuracy and interpretation of the results for PNCs.

When using electron microscopy, identifying well‐separated nonoverlapping nanoparticles was sometimes challenging and was not always well‐defined for very small PNCs <50 nm due to the low contrast resulting from their low electron density and the minimal sample thickness at the edges. PNCs >200 nm are flattened by capillary effects and surface forces. Therefore, the cryo‐TEM‐based size distribution of PNCs <50 and >200 nm should be interpreted with caution. Therefore, adaptation of the OECD guideline 125 [23] may be required for PNCs. For instance, the detection of PNCs <50 nm may require techniques such as Cryo‐TEM.

### 4.4 Warning Against the Use of Model PNC for Risk Assessment Studies

Discrepancies between suspensions produced at the lab scale and commercial scale, as well as the impact of the AS on PNC particle size and colloidal properties, indicate that using model PNCs may lead to inaccurate conclusions. We thus recommend using the final commercial PNC suspensions to generate reliable data and support robust exposure and (eco)toxicology assessment.

In conclusion, considering the importance of reliable size characterization to support risk assessment, it is clear that there is a need for more research to support the development of multimethod approaches applicable to PNCs and for the development or adaptation of OECD guidelines to suit the diversity of PNCs used across food, agricultural, and pharmaceutical sectors.

## Author Contributions

D.M. synthesized in‐house PNCs, performed experiments, analyzed data, and prepared the original draft. M.G. and T.T. collected and interpreted the DCS data. J.T.S. contributed to the overall supervision and critical revisions of the article. E.M.L. and M.K. contributed to the conceptualization, critical revisions, and overall supervision.

## Conflicts of Interest

The authors declare no conflicts of interest.

## Supporting information

Synthesis of in‐house PNCs, cryo‐TEM images illustrating the artefacts, DLS analysis data, PTA data and DSC analysis data can be found in the Supporting Information.

## Data Availability

The data supporting the findings of this study are available in the Supporting Information.
