# Supplementary material for: Particle Size Measurement of Organic Nanocarriers: A Case Study Using Polyacrylate‐Based Polymer Nanocarriers
Source: Small Sci. 2026 Oct 5;6(10):e70415. doi: 10.1002/smsc.70415 (PMC13635878; doi:10.1002/smsc.70415)
Supplement: Supplementary file 1 [file SMSC-6-e70415-s001.pdf]

Supporting Information for

Particle size measurement of organic nanocarriers: a case study using polyacrylate-based polymer nanocarriers

Dona Manayath, Monica Granetto, Tiziana Tosco, Jadranka Travas-Sejdic, Erin M. Leitao, Melanie Kah**

CryoTEM and SEM comparison

Dry-state high vacuum SEM imaging was conducted in all the PNC formulations and compared with the cryo-TEM images. High vacuum SEM dry state imaging was carried out using a Zeiss Gemini 460 microscope (Carl Zeiss AG, Germany) at an acceleration voltage of 1kV. A secondary electron detector was used for image generation. Prior to imaging, all the PNC formulations were filtered through 0.1 µm gold-coated 10 nm Nucleopore PC filters and coated with 3 nm of Platinum using EM ACE600 sputter coater (Leica Microsystems GmbH, Wetzlar, Germany).

The Cryo-TEM images were comparable to the images obtained from high-vacuum dry-state SEM images. However, the particle size observed in dry state SEM images was larger and approximately three times higher than that of the cryo-TEM images. This variation could be due to changes in the agglomeration behavior and surface characteristics of the PNCs under dry state conditions, confirming the importance of hydrated state characterization.

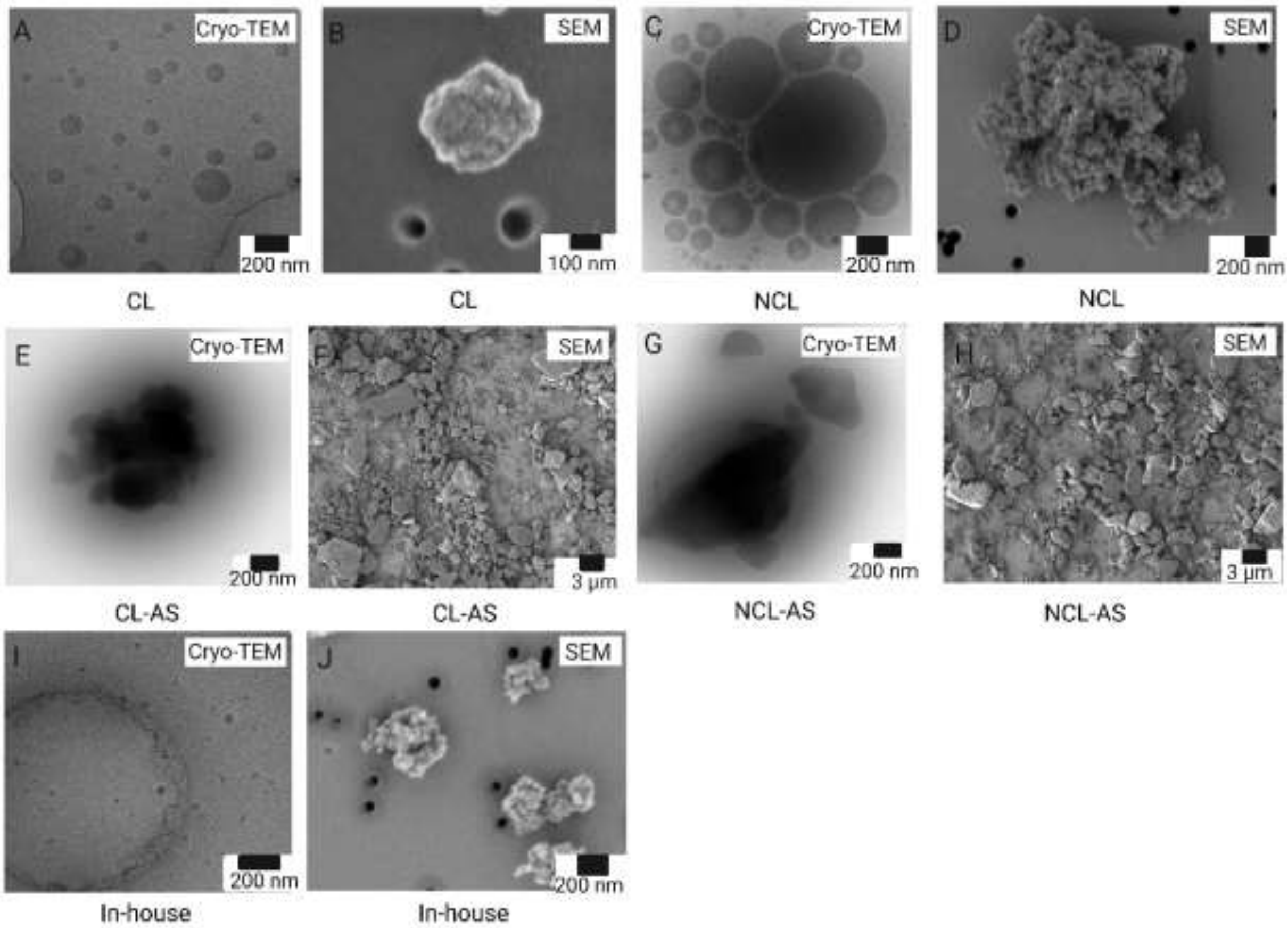

Figure S1. Cryo-TEM and high-vacuum SEM images of polymer nanocarriers (PNCs). **A)** and **B)** show the cryo-TEM and dry state high vacuum SEM images of crosslinked PNCs (CL), respectively. **C)** and **D)** correspond to the non-crosslinked PNCs (NCL), **E)** and **F)** represent the AS-loaded CL PNCs (CL-AS), while **G)** and **H)** show the AS-loaded NCL PNCs (NCL-AS). The high-vacuum SEM images of CL-AS and NCL-AS (**F** and **H**) confirm AS-induced agglomeration and deviation from spherical morphology. **I)** and **J)** present the cryo-TEM and dry-state high-vacuum SEM images of in-house PNCs.

Transmission Electron Microscopy

The conventional TEM images of empty CL PNCs were compared with the cryo-TEM. Compared to the cryo-TEM, small PNCs < 100 nm were challenging to observe using the continuous thin carbon grid under high vacuum conditions. Therefore cryo-TEM was used for further analysis.

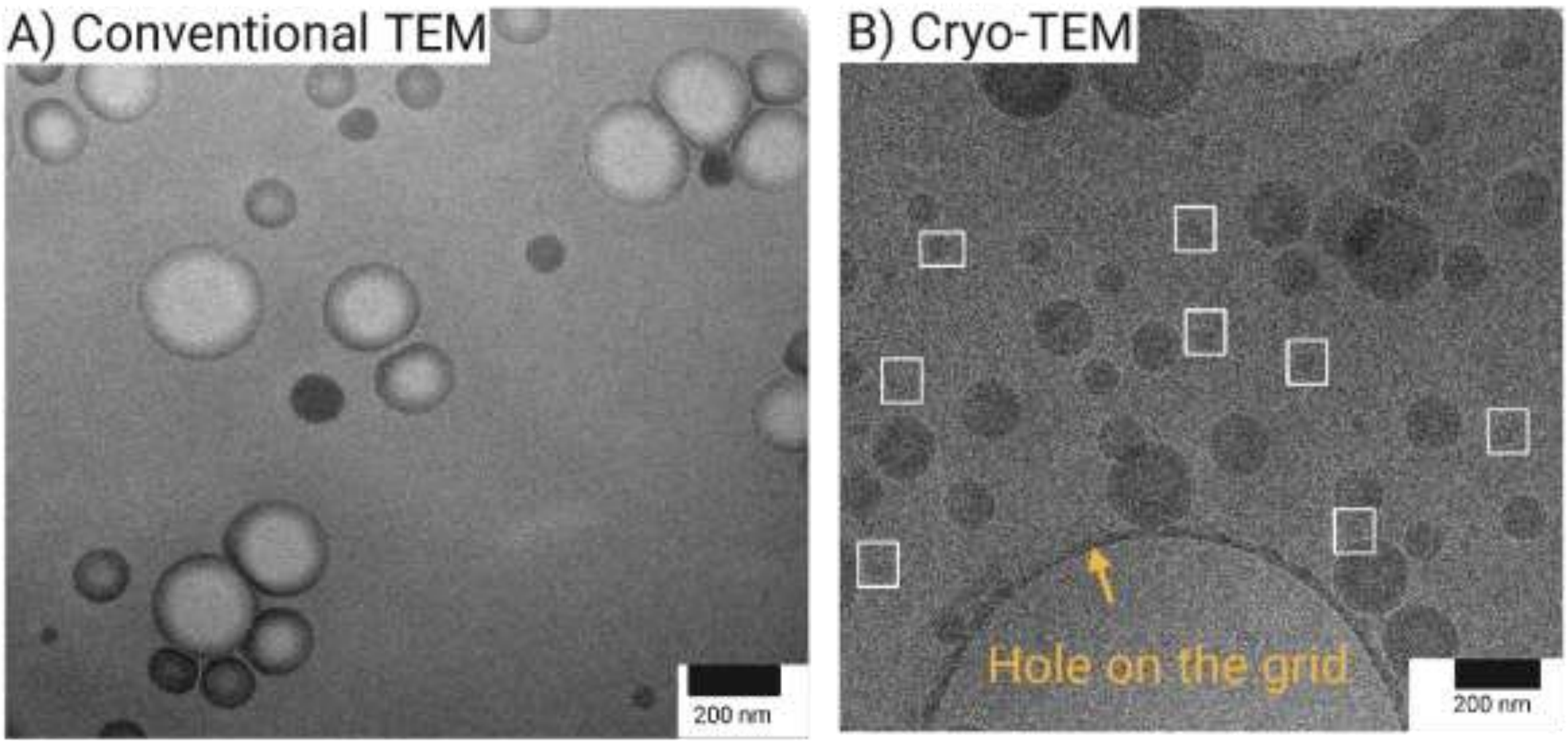

Figure S2. A) conventional TEM and **B)** cryo-TEM images of CL-PNCs. Here the particles in marked regions are less than 50 nm were not found in conventional TEM imaging.

TEM images showed artefacts from vitrified water due to crystalline ice formation. These are usually seen due to inadequate cooling or contact with atmospheric moisture, resulting in the formation of cubic ice crystals (**Figure S3A** and **B**). To avoid ice contamination, sample vitrification and cryo sample holder insertion should be completed within minutes.[1] In addition, due to inadequate cooling or improper grid clamping, the freeze-drying artefacts can be created under high vacuum conditions.[1] As a result of the freeze drying, bright and empty holes are created on the grid (**Figure S3C**). Moreover, the electron beam can cause damage to the soft PNC sample (**Figure S3D** and **E**). In summary, it is important to view PNCs through TEM in overview mode under low-dose conditions to prevent electron beam damage. Imaging can be performed using either high-dose quick capture mode or in low-dose conditions, depending on the electron beam stability of the PNCs of interest.

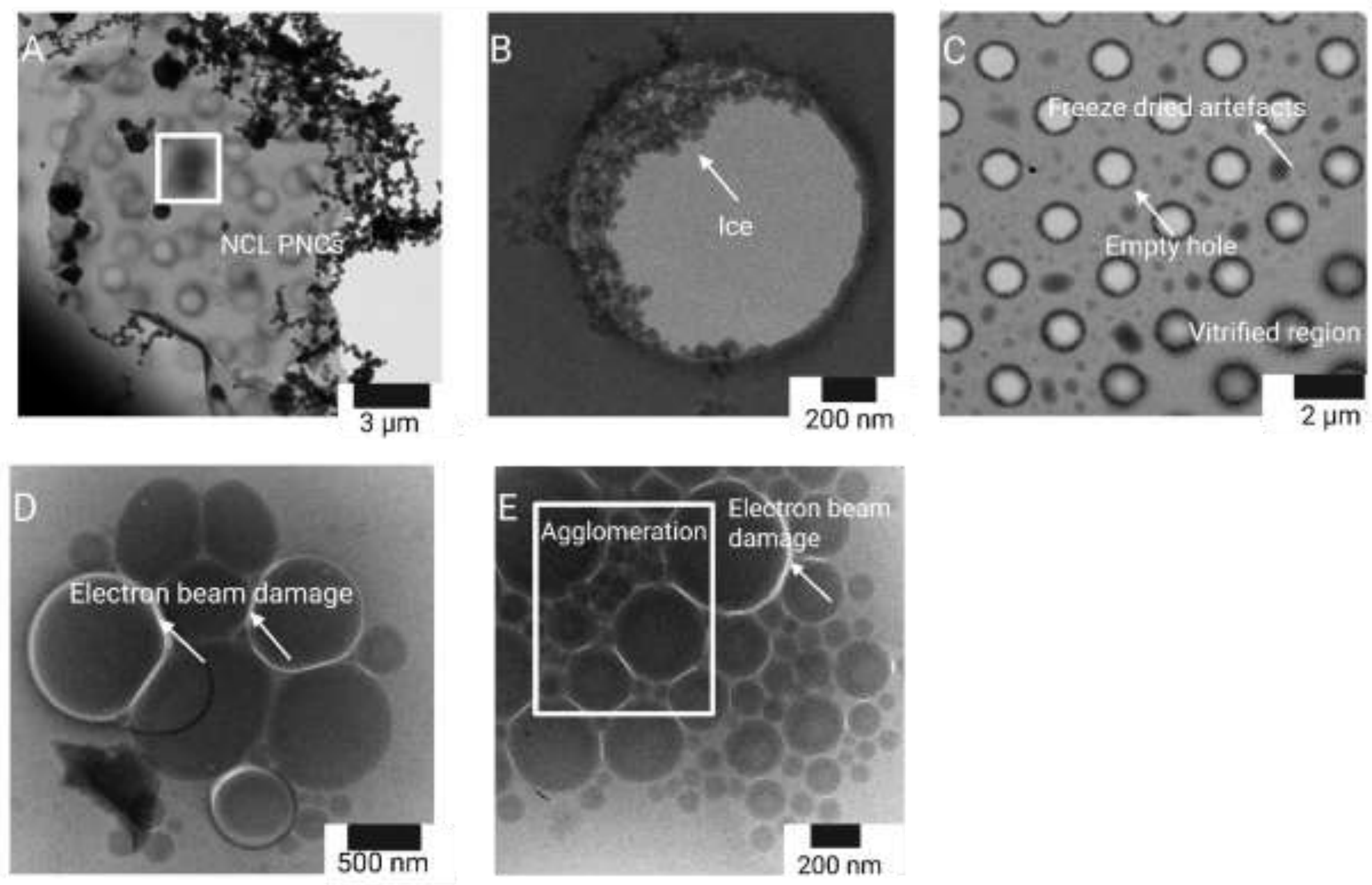

Figure S3. TEM images of various artefacts: **A)** and **B)** represent the formation of crystalline ice on the grid due to the inadequate cooling of the samples or due to contact with atmospheric moisture during sample preparation. Here, all the regions except the marked area as NCL PNCs, are crystalline ice artefacts. **C)** Represents the difference between the freeze-dried region and the vitrified region on a TEM grid surface. The freeze-drying results in artefacts on the grid surface due to improper grid clamping or inadequate cooling inside the cryo holder. **D)** Represents the electron beam damage of the NCL PNCs, while exposing the NCL PNCs for more than a few seconds, and **E)** represents the agglomeration found in NCL PNCs and the electron beam damage in large NCL PNCs.

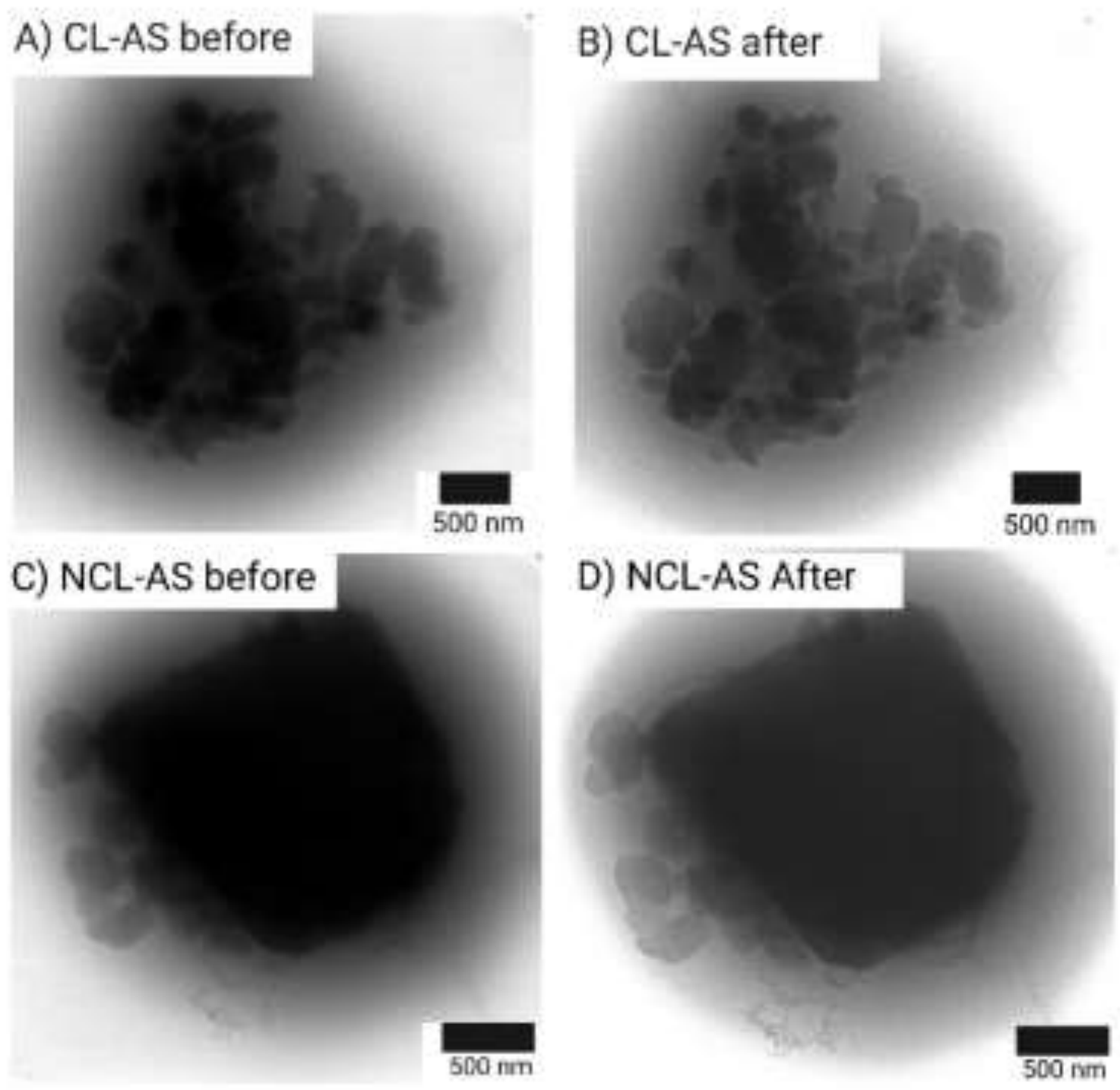

Figure S4. Cryo-TEM images of loaded PNCs before and after contrast enhancement using ImageJ: **A, C)** original images and **B, D)** corresponding contrast-enhanced images of CL-AS and NCL-AS PNCs, respectively.

Log-normal fit derived particle size measurements

Due to the high polydispersity found in NCL PNCs, the particle size values obtained from the simple arithmetic mean were higher than the log-normal fit (**Table S1**).

Table S1. Arithmetic mean and log normal; fit derived particle size distributions were obtained from the Image J processed TEM images of CL, NCL, and in-house PNCs.

PNC Sample	Particle size distribution /nm		R-square value
	Arithmetic mean	log-normal distribution fit	
CL	97 ± 86	97 ± 2	0.997
NCL	177 ± 128	129 ± 4	0.988
In-house	48 ± 20	45 ± 0.1	0.998

DLS data analysis

All the PNC samples analyzed through DLS were polydisperse with considerable variations between the sample replicates, which affects the reproducibility of the measurements. According to the OECD test guideline 125,[2] obtaining a reproducible particle size distribution is important for characterising the nanomaterials.

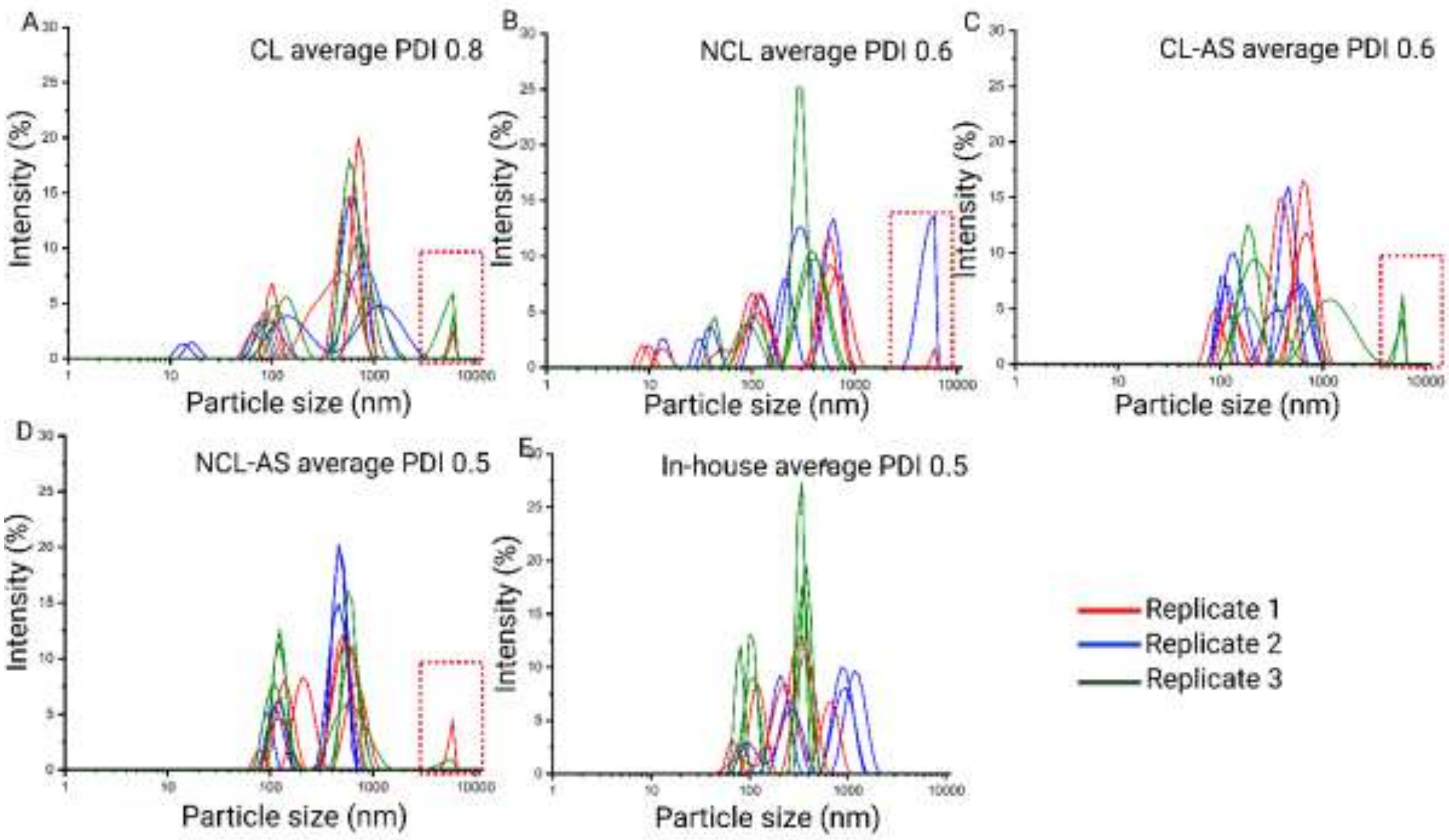

Figure S5. The intensity-based particle size distribution for three replicates of **A)** CL, **B)** NCL, **C)** CL-AS, **D)** NCL-AS, and **E)** in-house PNCs. The dotted red box indicates the tailings on the size distribution contributed by the large particles

NTA

The NTA of all PNC samples indicates polydispersity in each sample, where particles of different sizes are present and each replicate of the PNC sample results in a reproducible median hydrodynamic particle size distribution.

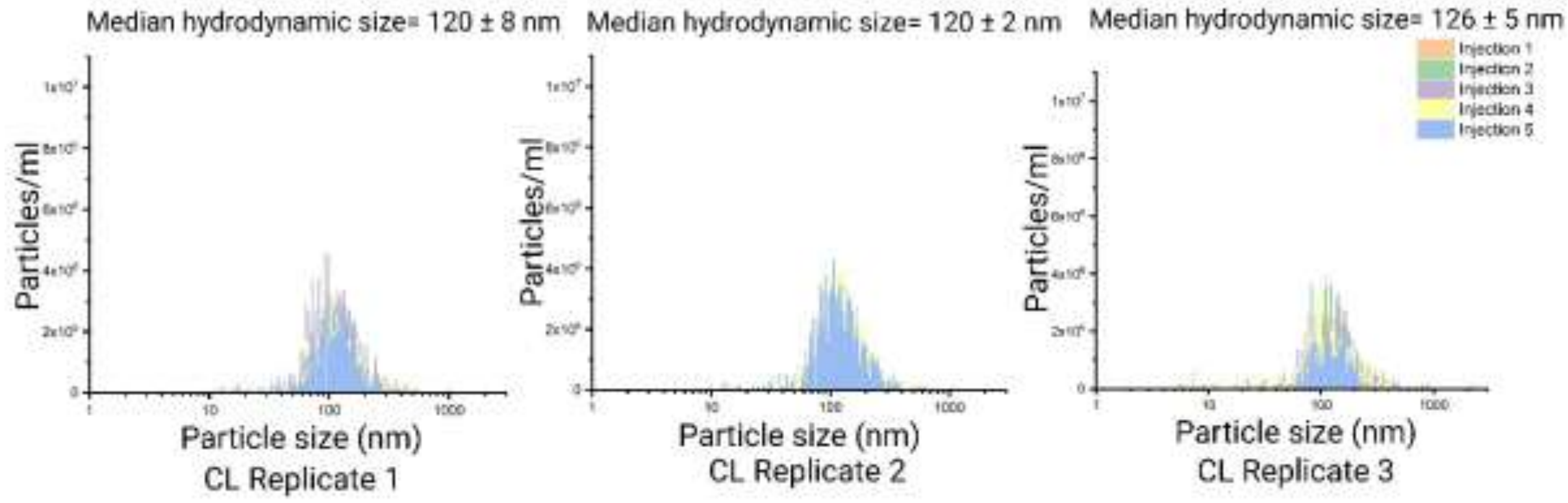

Figure S6. Particle size analysis of three replicates (left-right) of crosslinked PNCs (CL) using NTA with five injections and five measurements per replicate. The pre-acquisition sensitivity parameter is fixed at 80.

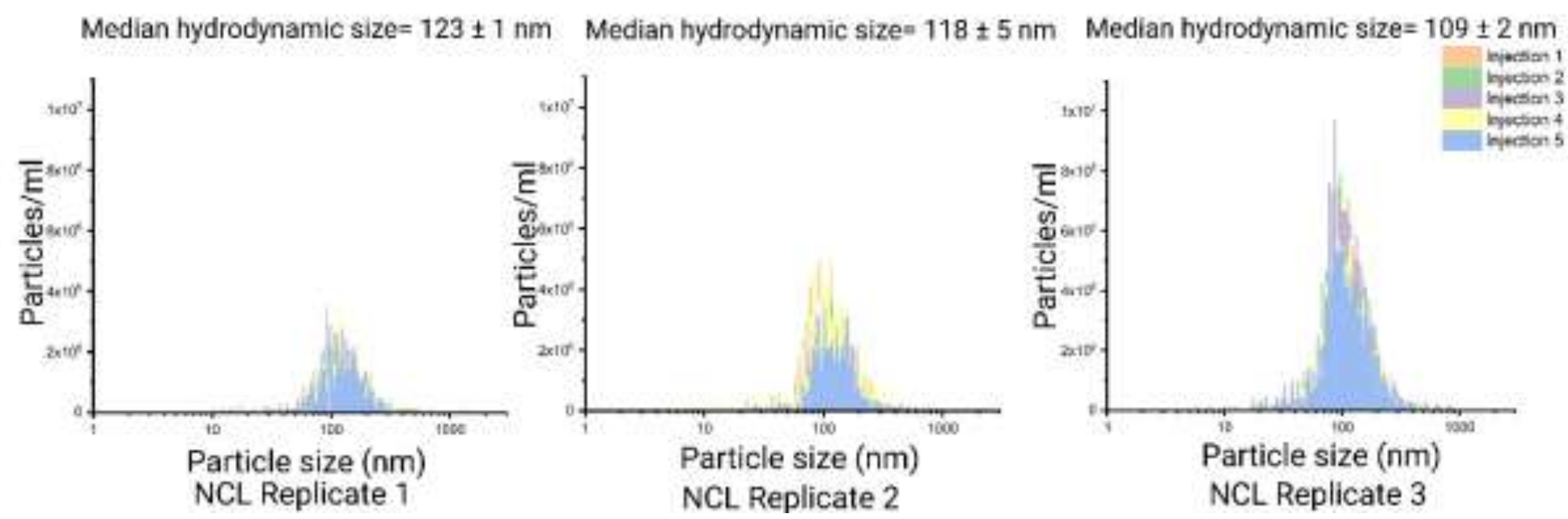

Figure S7. Particle size analysis of three replicates (left-right) of non-crosslinked PNCs (NCL) using NTA with five injections and five measurements per replicate. The pre-acquisition sensitivity parameter is fixed at 80.

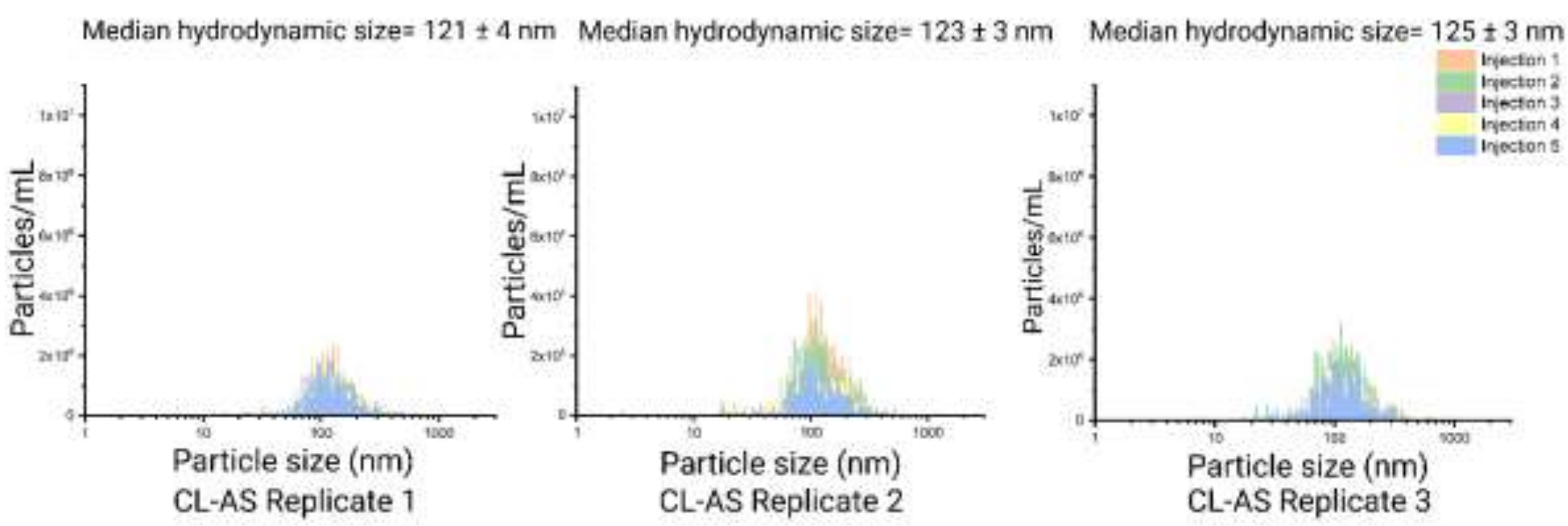

Figure S8. Particle size analysis of three replicates (left-right) of AS loaded crosslinked PNCs (CL-AS) using NTA with five injections and five measurements per replicate. The pre-acquisition sensitivity parameter is fixed at 80.

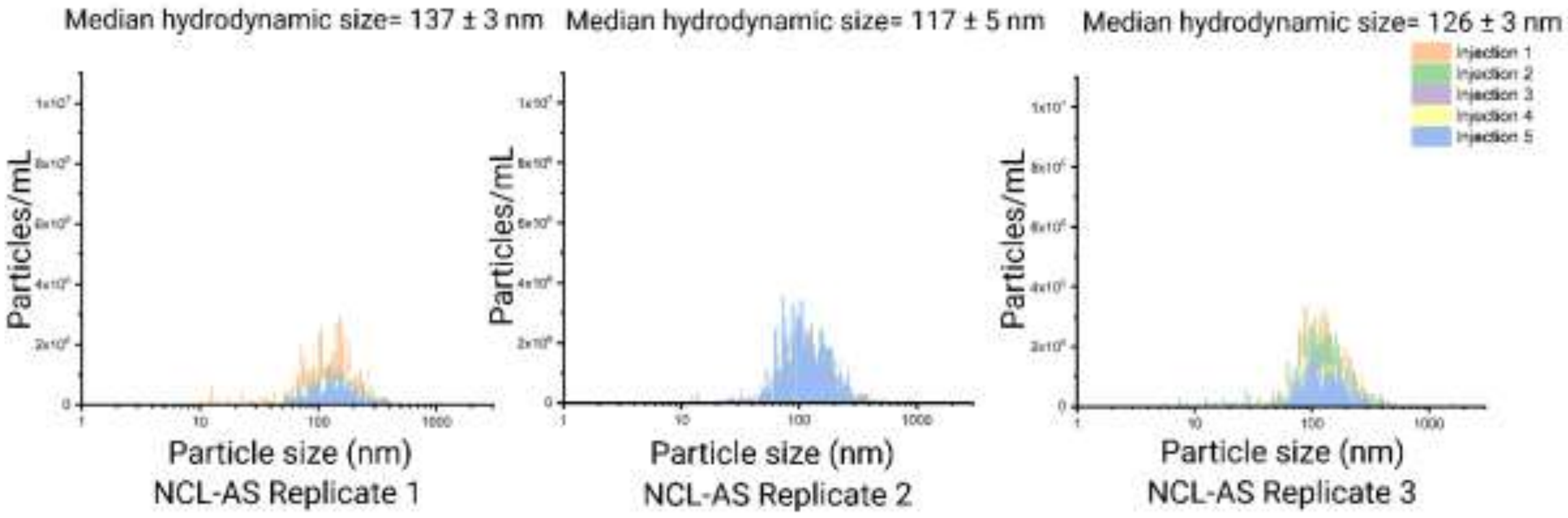

Figure S9. Particle size analysis of three replicates (left-right) of non-crosslinked PNCs with AS (NCL-AS) using NTA with five injections and five measurements per replicate. The pre-acquisition sensitivity parameter is fixed at 80.

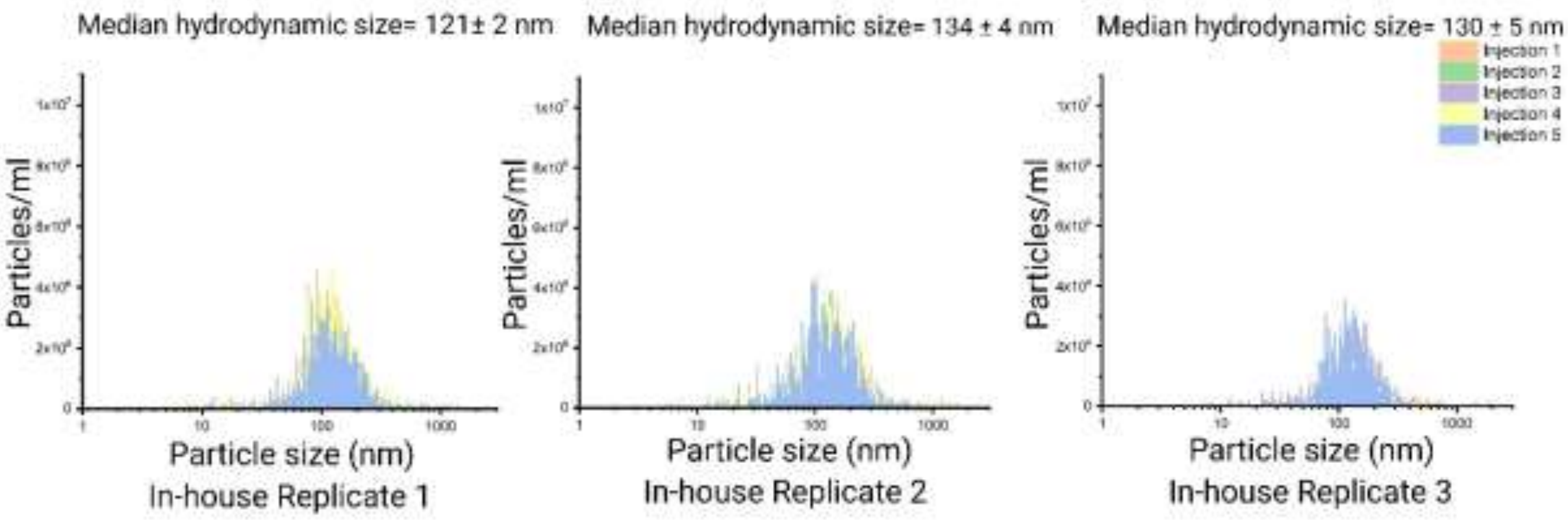

Figure S10. Particle size analysis of three replicates (left-right) of laboratory scale PNCs (in-house) using NTA with five injections and five measurements per replicate. The pre-acquisition sensitivity parameter is fixed at 80.

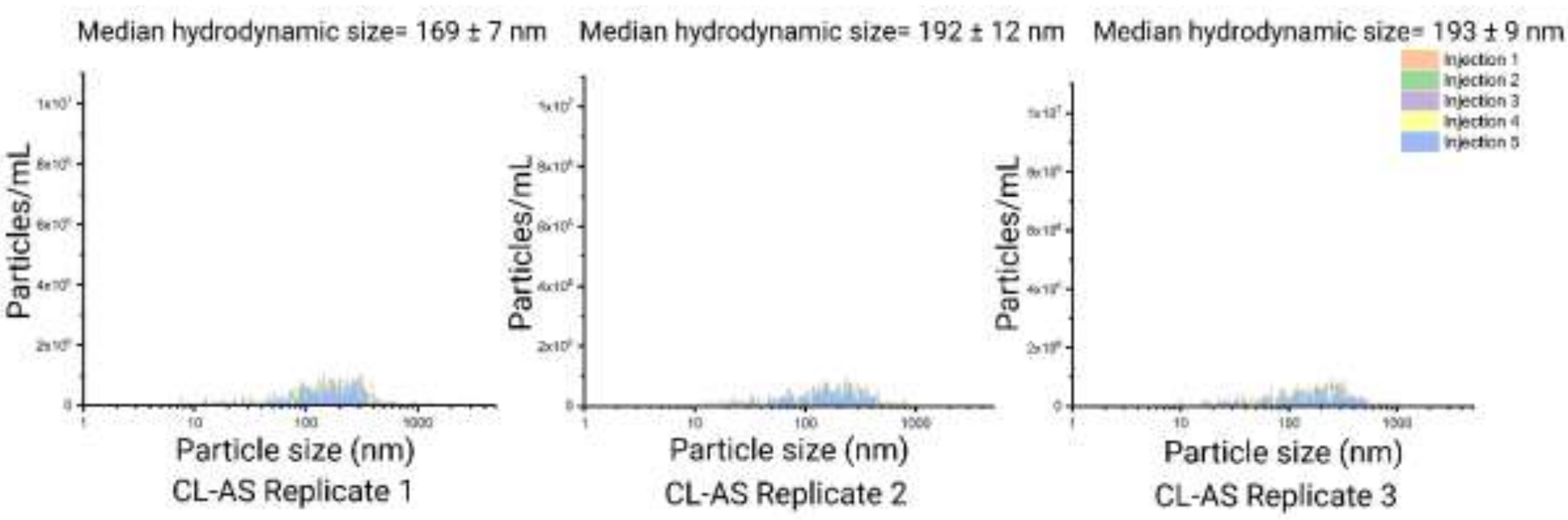

Figure S11. Particle size analysis of three replicates (left-right) of AS loaded crosslinked PNCs (CL-AS) using NTA with five injections and five measurements per replicate. The pre-acquisition sensitivity parameter is fixed at 60.

In **Figure S11** and **S13**, a significant reduction in particle concentration was observed when the sensitivity parameter was reduced to 60, indicating that a large number of small PNCs were excluded from the analysis. This is further supported by the live video frame acquired during the NTA analysis data acquisition (**Figure S12**).

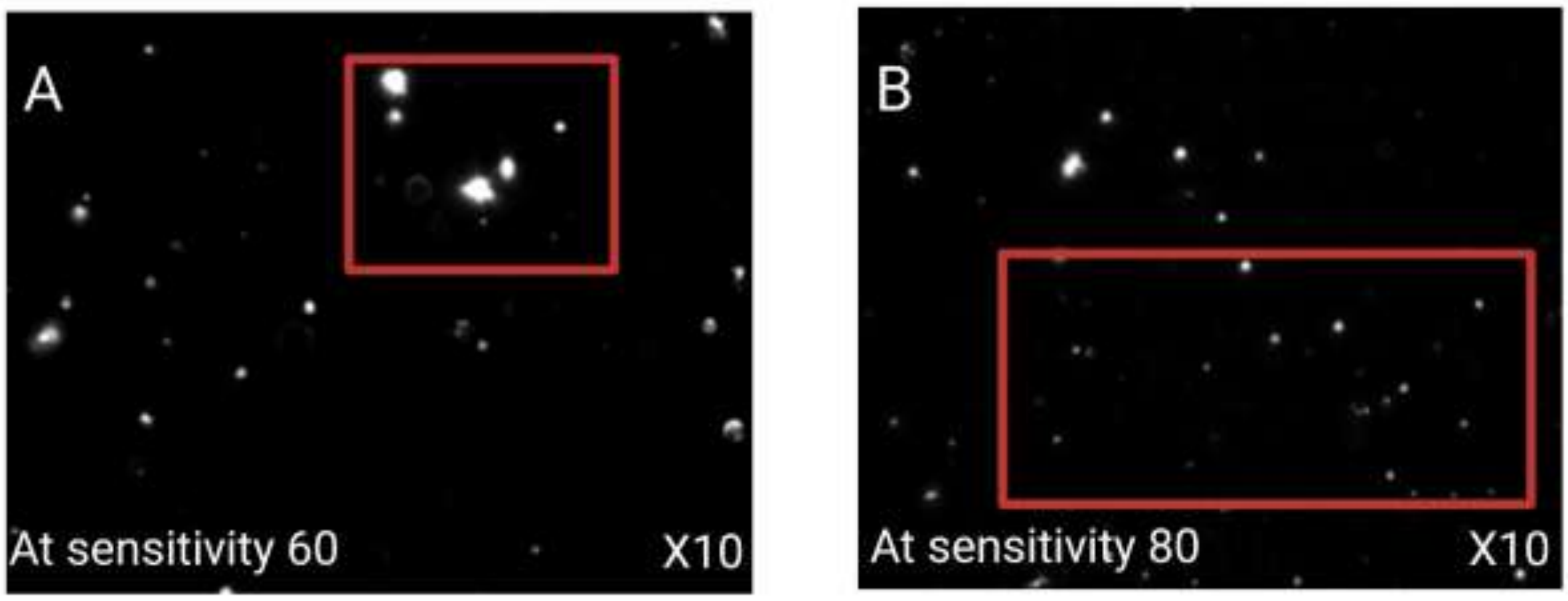

Figure S12. A) and **B)** represent NTA live video frame of the CL-AS particles tracked by a 90° laser scattering video microscope with 10x magnification, with sensitivity 60 and 80, respectively. Small PNCs are not detected in A at a sensitivity of 60, whereas at a sensitivity of 80, large particles are excluded, and more small particles are detected (regions marked in red).

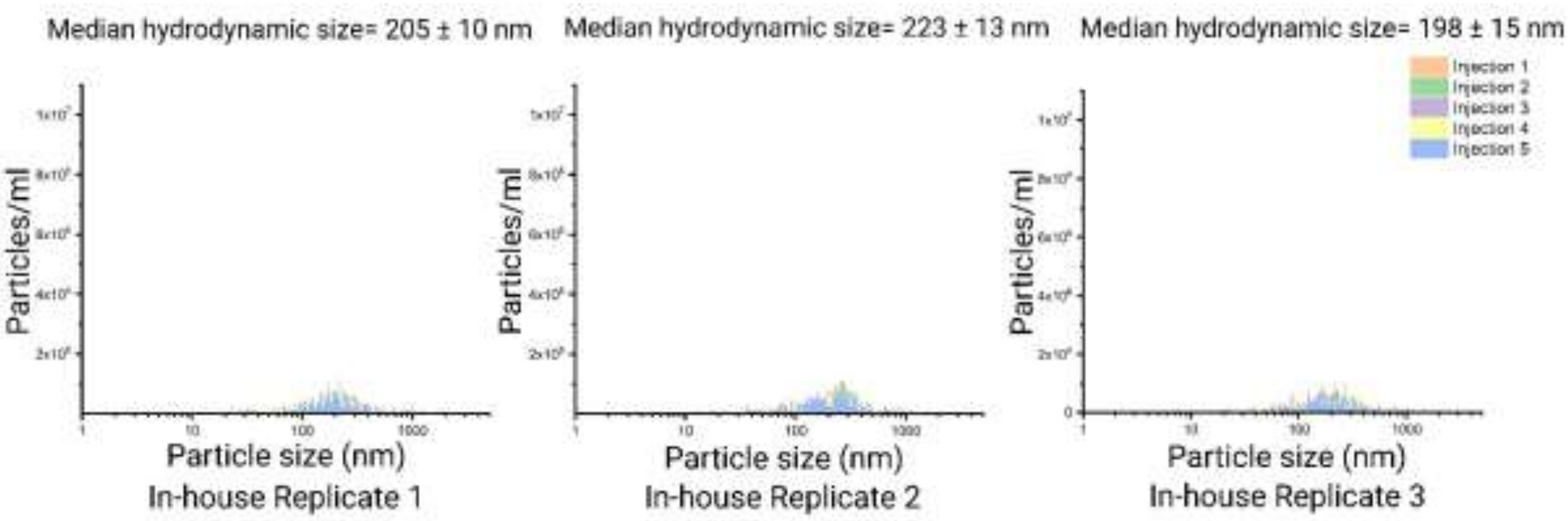

Figure S13. Particle size analysis of three replicates (left-right) of laboratory scale PNCs (in-house) using NTA with five injections and five measurements per replicate. The pre-acquisition sensitivity parameter is fixed at 60.

DCS analysis

In DCS analysis, the number-based particle number distribution and cumulant distribution (cumulative percentage of particles below a given size) are normalized over the maximum value and plotted against the particle size measurements.

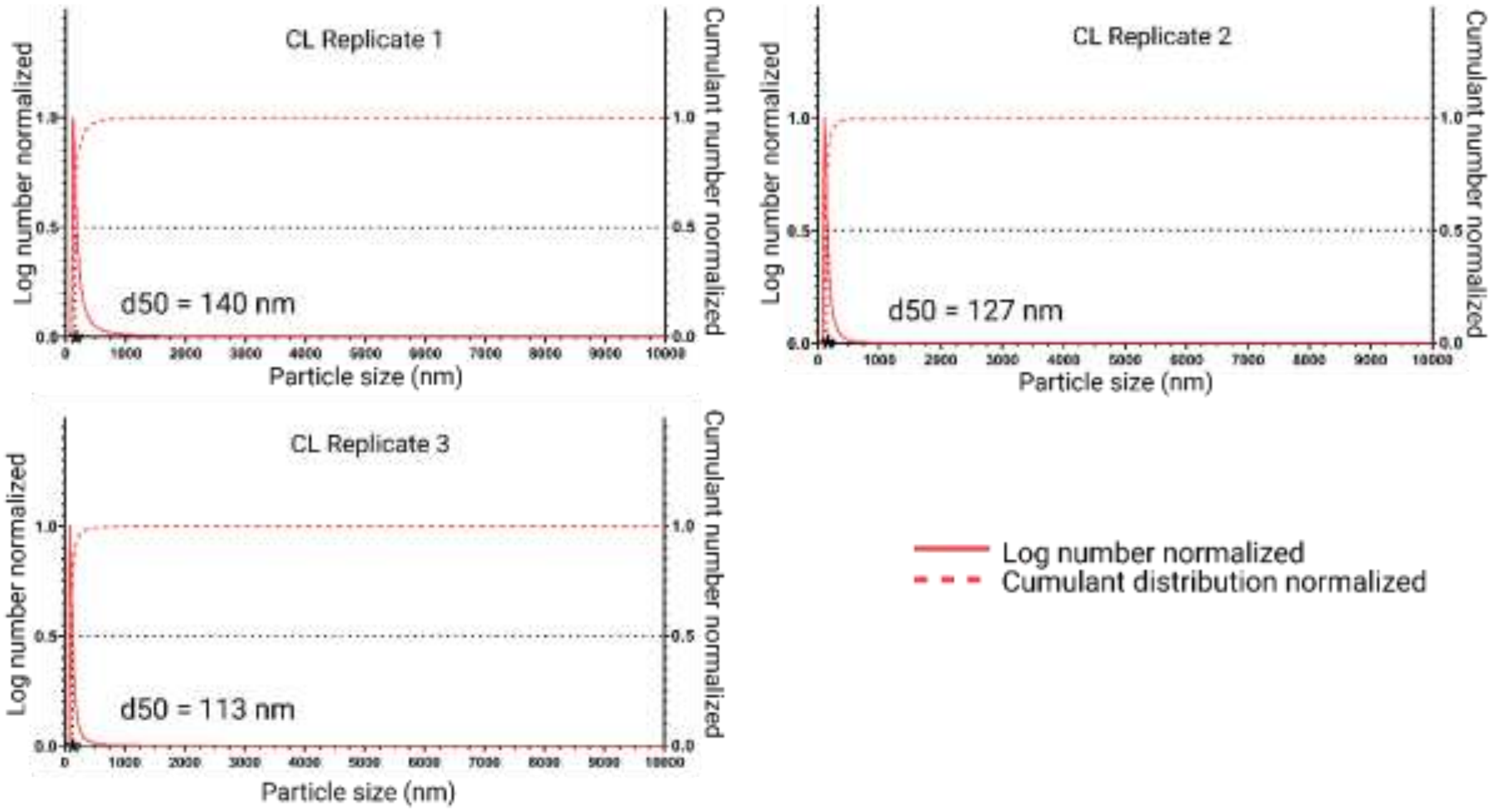

Figure S14. Particle size analysis of three replicates (left-right) of crosslinked PNCs (CL) CL PNCs using DCS.

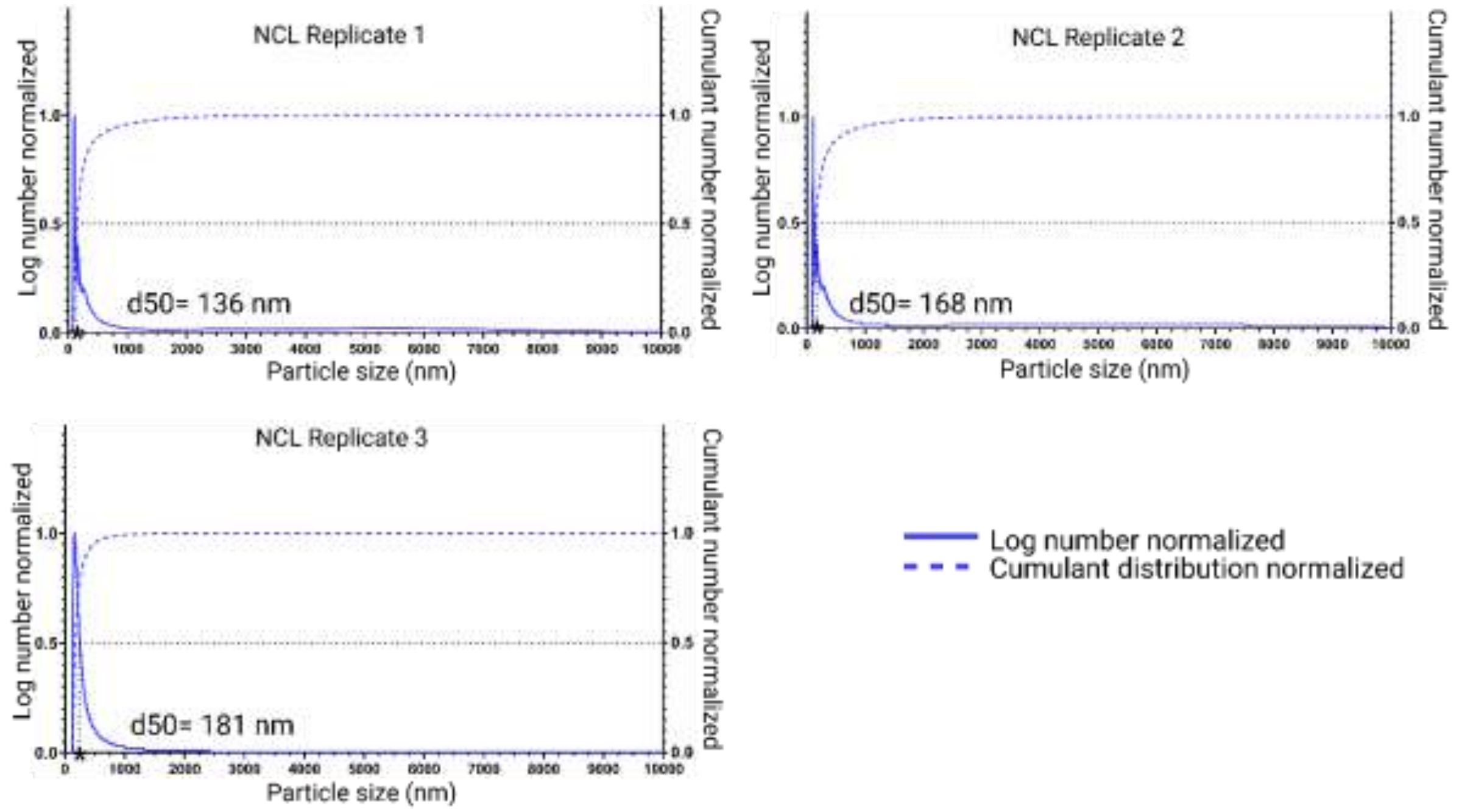

Figure S15. Particle size analysis of three replicates (left-right) of non-crosslinked PNCs (NCL)using DCS.

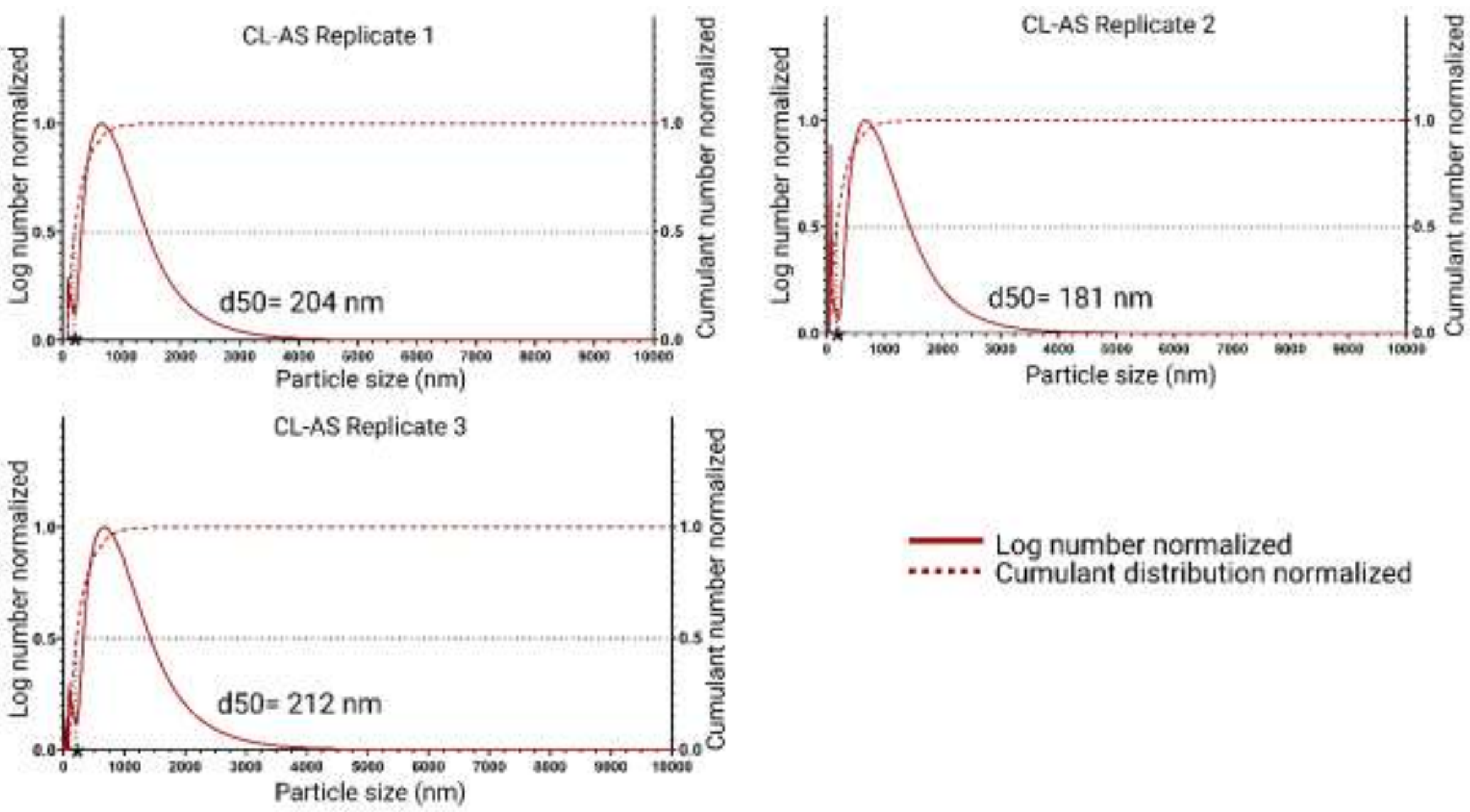

Figure S16. Particle size analysis of three replicates (left-right) of AS loaded crosslinked PNCs (CL-AS) using DCS.

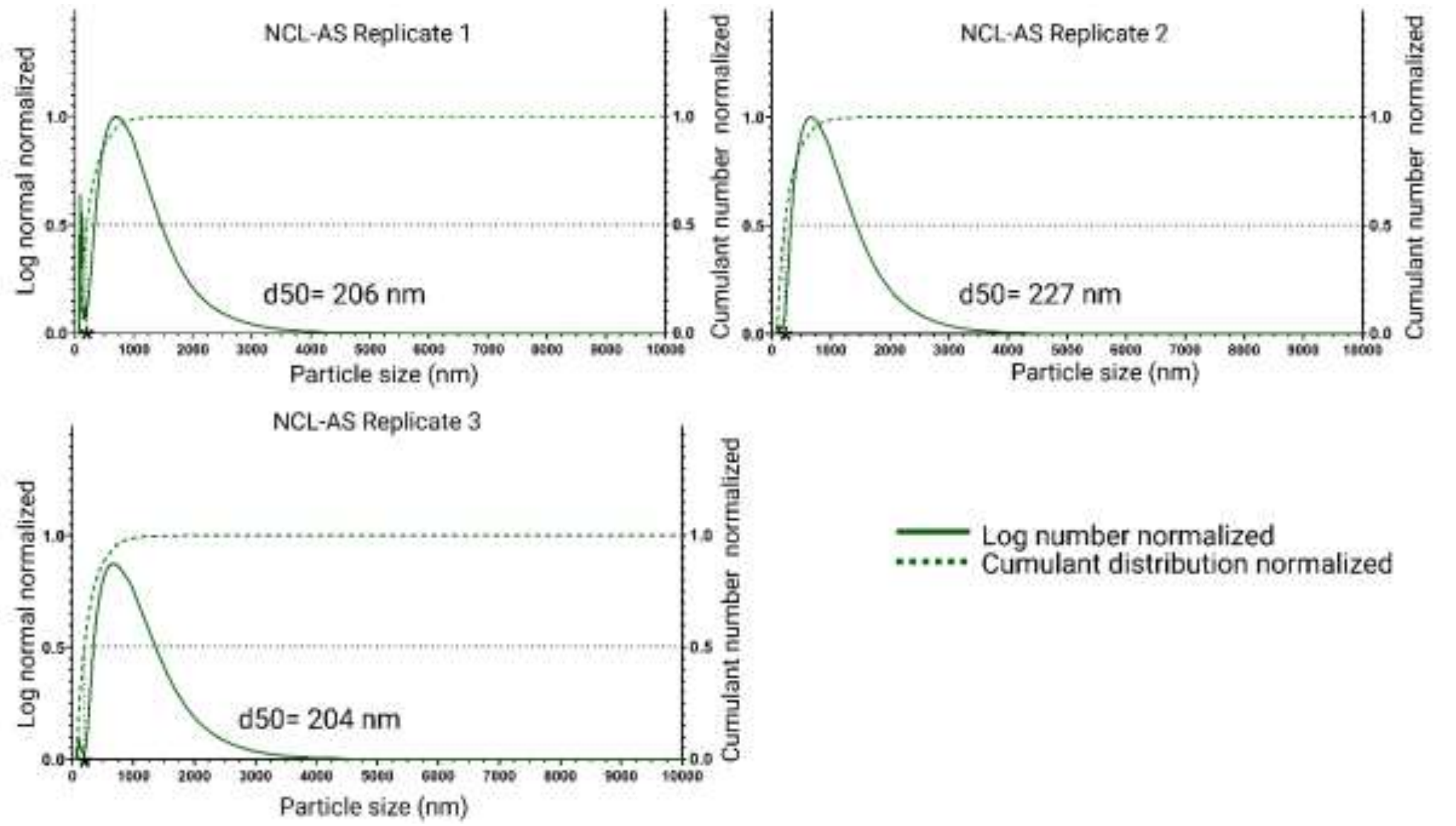

Figure S17. Particle size analysis of three replicates (left-right) of non-crosslinked PNCs with AS (NCL-AS) using DCS.

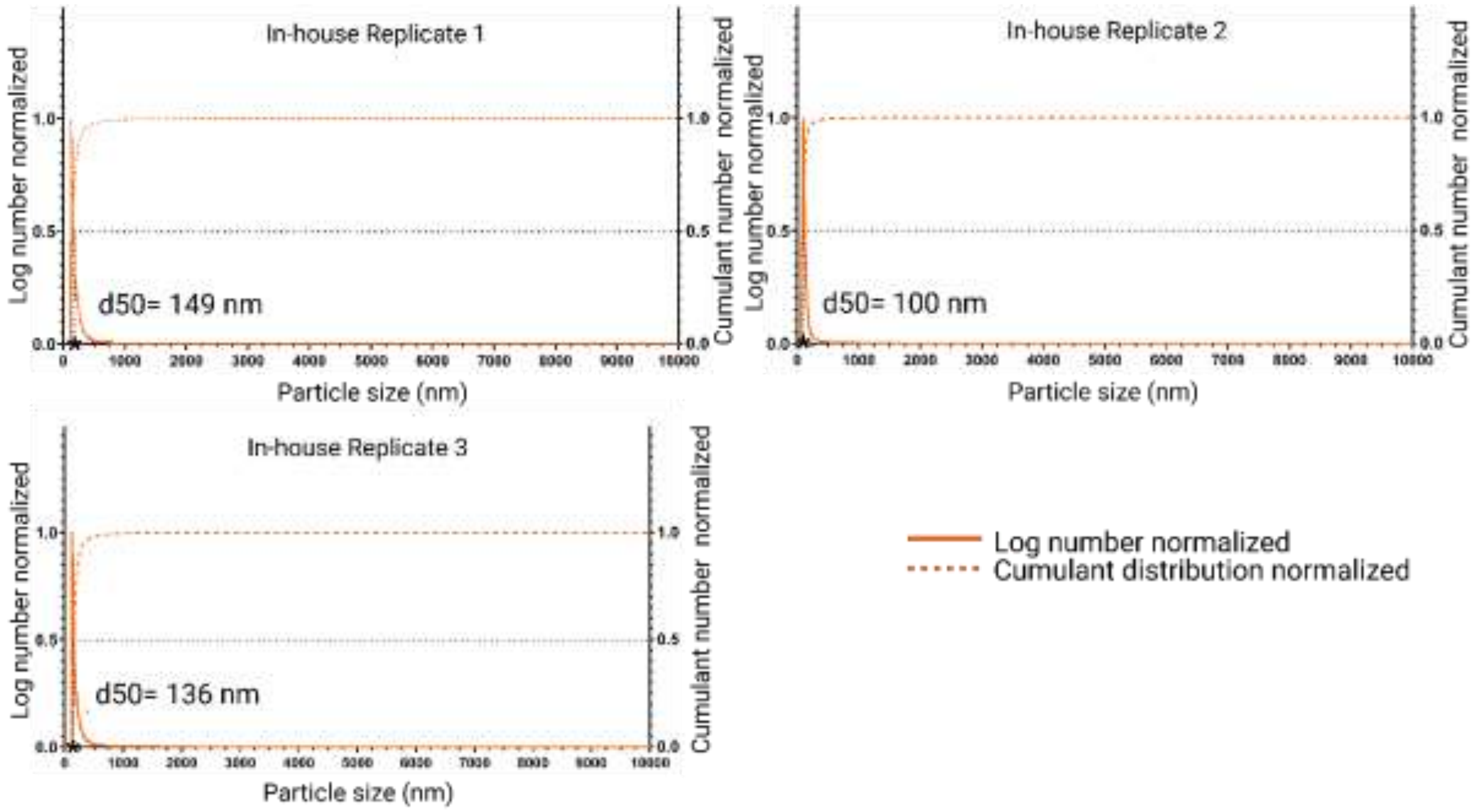

Figure S18. Particle size analysis of three replicates (left-right) of laboratory scale PNCs (in-house) using DCS.

References

(1) Kuntsche, J.; Horst, J. C.; Bunjes, H. Cryogenic Transmission Electron Microscopy (Cryo-TEM) for Studying the Morphology of Colloidal Drug Delivery Systems. *Int. J. Pharm.* **2011**, *417* (1–2), 120–137. https://doi.org/10.1016/j.ijpharm.2011.02.001.
(2) OECD. *Test No. 125: Nanomaterial Particle Size and Size Distribution of Nanomaterials*; Organisation for Economic Co-operation and Development: Paris, 2023.
